# Sequential generation of olfactory bulb glutamatergic neurons by Neurog2-expressing precursor cells

**DOI:** 10.1186/1749-8104-6-12

**Published:** 2011-04-05

**Authors:** Eleanor Winpenny, Mélanie Lebel-Potter, Maria E Fernandez, Monika S Brill, Magdalena Götz, Francois Guillemot, Olivier Raineteau

**Affiliations:** 1 Cambridge Centre for Brain Repair, Robinson Way, CB2 0PY Cambridge, UK; 2 Division of Molecular Neurobiology, Medical Research Council - National Institute for Medical Research, NW7 1AA London, UK; 3 Brain Research Institute, University of Zürich/ETHZ, 8057 Zürich, Switzerland; 4 Department of Physiological Genomics, Institute of Physiology, Ludwig-Maximilians University Munich, 80636 Munich, Germany; 5 Institute for Stem Cell Research, Helmholtz Zentrum München, 85764 Munich, Germany; 6 Munich Center for Integrated Protein Science CiPSM, 81377 Munich, Germany

## Abstract

**Background:**

While the diversity and spatio-temporal origin of olfactory bulb (OB) GABAergic interneurons has been studied in detail, much less is known about the subtypes of glutamatergic OB interneurons.

**Results:**

We studied the temporal generation and diversity of Neurog2-positive precursor progeny using an inducible genetic fate mapping approach. We show that all subtypes of glutamatergic neurons derive from Neurog2 positive progenitors during development of the OB. Projection neurons, that is, mitral and tufted cells, are produced at early embryonic stages, while a heterogeneous population of glutamatergic juxtaglomerular neurons are generated at later embryonic as well as at perinatal stages. While most juxtaglomerular neurons express the T-Box protein Tbr2, those generated later also express Tbr1. Based on morphological features, these juxtaglomerular cells can be identified as tufted interneurons and short axon cells, respectively. Finally, targeted electroporation experiments provide evidence that while the majority of OB glutamatergic neurons are generated from intrabulbar progenitors, a small portion of them originate from extrabulbar regions at perinatal ages.

**Conclusions:**

We provide the first comprehensive analysis of the temporal and spatial generation of OB glutamatergic neurons and identify distinct populations of juxtaglomerular interneurons that differ in their antigenic properties and time of origin.

## Background

The development of the olfactory bulb (OB) is traditionally believed to occur in two phases. The initial stages of OB development show many similarities to the development of the neocortex. The first cells to be born are the glutamatergic projection neurons, the mitral and tufted cells of the OB, beginning at embryonic day (E)11 [1,2]. The mitral cells are produced first, followed by the tufted cells in an inside-out sequence, with superficial tufted cells the last to be born. At this time, newborn OB neurons are born in the ventricular zone (VZ) of the OB region, from radial glia, as in other cortical regions. Newborn cells migrate radially to their final positions, where they differentiate.

As the production of excitatory projection neurons continues and begins to slow, a second developmental phase starts with the arrival of GABAergic interneurons in the OB. Whilst some interneurons have an intrabulbar origin [3], most of them emanate first from the lateral ganglionic eminence [4], and then from the rostral migratory stream (RMS) [5] and subventricular zone (SVZ) [6]. The peak of interneuron production is at perinatal ages, and continues throughout adult life [7-9].

During development, neuronal specification relies on the differential expression of distinct transcription factors. The basic helix-loop-helix (bHLH) transcription factor Neurog2 has typically been associated with the development of glutamatergic neurons [10-14]. Neurog2 participates in a cascade of transcription factors comprising Pax6, Tbr2 and Tbr1, which together promote the generation of glutamatergic neurons in both the cortex and the hippocampus. In the developing cortex, Neurog2 has been proposed to be directly responsible for the activation of a cortical glutamatergic transcriptional pathway and the repression of GABAergic transcription factors such as Dlx2 [14]. At later stages, Neurog2 is believed to act in sequence with Mash1 to regulate the transition of neuronal precursors from the VZ to the SVZ [15].

Various classes of glutamatergic OB neurons have been described: mitral and tufted cells, which project and transfer information to a number of extrabulbar areas in the brain [16]; and glutamatergic interneurons of the glomerular layer (GL), which are subdivided into external tufted cells [17] and short-axon cells [18]. These two subtypes of neurons show intrabulbar axonal projections and play important roles in the processing of olfactory information [17-20]. Here, we use an inducible genetic fate mapping of Neurog2 precursors to study the temporal profile by which glutamatergic neuronal subtypes are generated. We provide a comprehensive analysis of the temporal generation of OB glutamatergic neurons and identify distinct populations of juxtaglomerular interneurons that differ in their antigenic properties and time of origin. Moreover, our results suggest that some glutamatergic juxtaglomerular neurons originate from extrabulbar regions at perinatal ages.

## Results

### Expression pattern of Neurog2, Pax6 and Tbr1/2 during olfactory bulb development

**Figure 1:**
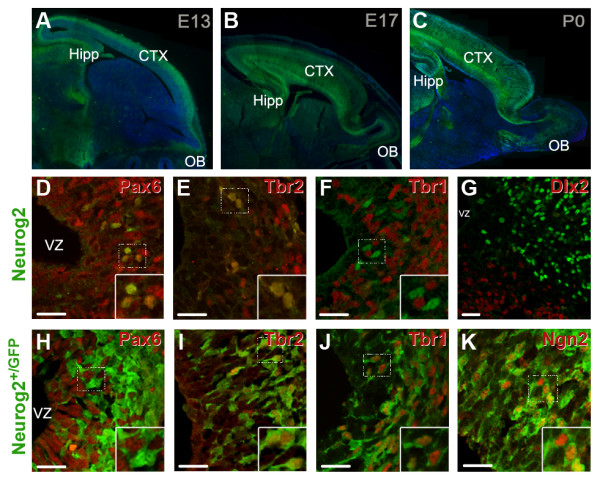
**Spatial and temporal expression of Neurog2 in the developing mouse olfactory bulb**. (**A-C) **Overview of the distribution of Ngn2GFP expression at E13.5 and E17.5 and postnatal day 0 in the cortex, SVZ and OB. Rostral is to the right of the image. **(D-G) **Co-localization of Neurog2 with Pax6, Tbr2, Tbr1 and Dlx2 at E13.5. Note the high co-localization between Neurog2, Pax6 and Tbr2, and the minor co-localization between Neurog2 and Tbr1. Note that Neurog2 and Dlx2 were observed in different compartments of the developing OB, with Dlx2-positive cells located more ventrally and caudally than Neurog2-positive cells. In the region of overlap, although rare Neurog2+ cells expressed low levels of Dlx2, no significant co-localization could be observed. **(H-K) **Co-localization of Neurog2^+/GFP ^with Pax6, Tbr2, Tbr1 and Neurog2 at E13.5. Little co-localization is seen between Neurog2^+/GFP ^and Pax6; however, high co-expression of Neurog2^+/GFP ^and Tbr2 and Tbr1 reveals that these markers are expressed in the same population of cells. Inserts show a higher magnification of the region surrounded by a dotted box. All photomicrographs in (D-K) show the same orientation as (A), with the VZ visible on the left and the OB parenchyma present on the right. Scale bar: 50 μm. CTX, cortex; Hipp, hippocampus; OB, olfactory bulb; VZ, ventricular zone.

We first visualized the expression of Neurog2 at different time points by using heterozygous *Neurog2*^+/GFP ^mice, in which green fluorescent protein (GFP) is inserted into one copy of the *Neurog2 *gene (Figure 1A-C). Immunostaining for Neurog2 confirmed the restricted expression of GFP to Neurog2-positive (Neurog2(+)) progenitors, confirming previous results [10,21] (Figure 1K). However, as the GFP degrades less rapidly than the Neurog2 protein, GFP revealed the immediate progeny of the Neurog2(+) cells. We investigated the pattern of *Neurog2*^+/GFP ^expression in the OB at three developmental stages: E13.5, that is, shortly after the formation of the OB has begun; E17.5; and postnatal day (P)0 [1]. At all three time points there was high expression of *Neurog2*^+/GFP ^in distinct regions of the developing forebrain (Figure 1A-C). We observed that whereas the GFP cells at E13.5 appear to be born in the presumptive OB, by E17 many cells are observed in the developing RMS. These cells express DCX (data not shown), suggesting that they migrate to the OB from more caudal ventricular regions.

In many regions of the brain, both during development and in the adult, specification of glutamatergic neurons has been associated with the sequential expression of transcription factors comprising Pax6, Neurog2, Tbr2 and Tbr1 [10,12]. All markers were expressed in the developing OB at E13.5, with partially overlapping patterns of expression. Comparison of the degree of overlap between these markers and Neurog2 (Figure 1D-F) or Neurog2^+/GFP ^(Figure 1H-J) revealed a similar sequential expression of these markers in the developing OB. We observed a radial pattern of expression of these transcription factors in the OB region, with the youngest cells found in the VZ expressing Pax6, followed by expression of Neurog2, Tbr2 and Tbr1 as the cells migrate outwards towards the OB periphery. While Neurog2 showed minimal co-localization with Tbr1, a larger number of Neurog2^+/GFP ^cells expressed this marker. Because of the longer half-life of GFP (see above), these results illustrate the expression of Tbr1 in the immediate progeny of Neurog2(+) progenitors. In contrast, both Neurog2(+) and Neurog2^+/GFP ^cells were negative for Dlx2, a homeodomain transcription factor expressed in GABAergic progenitors. Whilst both progenitor populations were present in the OB [3], they showed a clear spatial segregation, with Dlx2-progenitors showing a more caudal distribution than Neurog2(+) progenitors (Figure 1G).

### Fate mapping of Neurog2-expressing progenitors during olfactory bulb development

In order to determine more precisely the contribution of Neurog2(+) progenitors to developmental OB neurogenesis, we next used mice expressing a tamoxifen-inducible Cre recombinase under the *Neurog2 *promoter, that is, *Neurog2iCreERT2 *(see Materials and methods). *In situ *hybridization and immunodetection of Cre and Neurog2 revealed an overlapping pattern of the two markers (Additional file 1). These mice were crossed with RosaYFP or TaukiGFP reporter mice. The second of these reporter mice expresses a membrane bound form of GFP (see Materials and methods), allowing one to visualize the detailed morphology of the recombined cells [22]. Tamoxifen injections at E13.5, E17.5 and P0 induced a rapid recombination in a small number of Neurog2 expressing cells, observable as early as 24 hours post-injection (Additional file 2). At this short time point, immunodetection of Tbr2 and Dlx2 in the GFP(+) cells (100% and 0% co-localization, respectively; 47 cells analyzed; Additional file 2) supports the restricted expression of the Cre recombinase, and the efficient labeling of the Neurog2 cell lineage.

**Figure 2:**
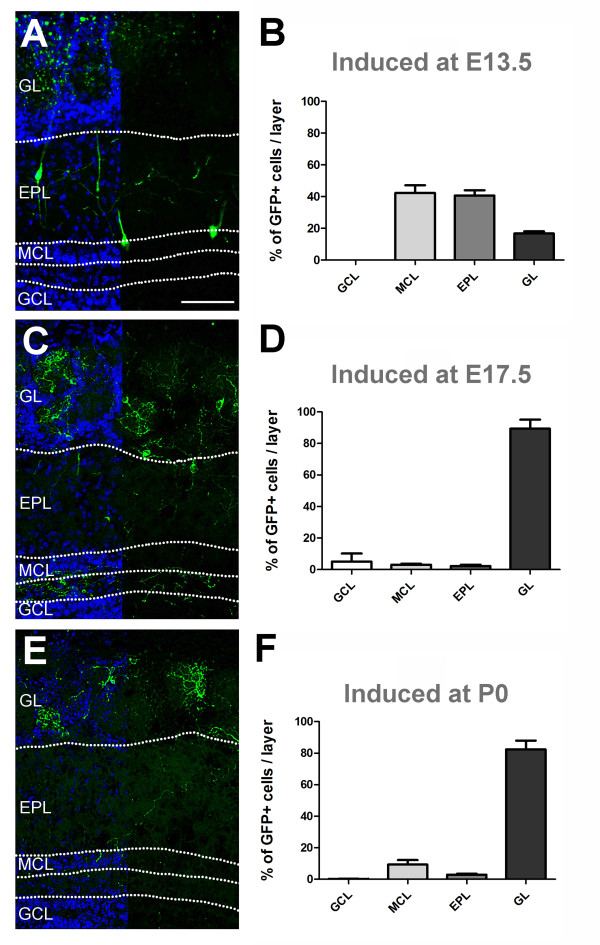
**Neurog2 progeny populate distinct OB layers during embryonic development**. **(A,C,E) **Overview of the distribution of GFP-positive cells in the OB of P21 *Neurog2CreERT2 *× TaukiGFP mice. The recombination was induced at E13.5 (A), E17.5 (C) and at birth (E) by a single injection of tamoxifen. DAPI counterstaining (blue) on the left side of the photomicrograph visualizes the distinct OB layers. **(B,D,F) **Quantification of the percentage of Neurog2-derived GFP-positive cells observed in the OB layers following tamoxifen injection at E13.5 (B), E17.5 (D) and at birth (F). Scale bar: 100 μm. EPL, external plexiform layer; GL, glomerular layer; GCL, granular cell layer; MCL, mitral cell layer. Error bars indicate S.E.M.

We next investigated the fate of E13.5, E17.5 and P0 Neurog2(+) progenitors by quantifying GFP(+) cells in the OB layers at P21 (Figure 2). In all groups of mice, the vast majority of GFP(+) cells were observed in the mitral cell layer (MCL), external plexiform layer (EPL) and GL, and only a very small number was observed in the granule cell layer. Animals injected at E13.5 had GFP cells with cell bodies in the MCL (42.3 ± 4.8%) and EPL (40.6 ± 3.3%). At this early embryonic time point, only a small population of GFP(+) cells was observed in the GL (16.7 ± 1.3%; Figure 2A,B; 1,192 GFP(+) cells analyzed in 3 animals). Measurement of the cell body diameter revealed a significant decrease from the MCL to the GL (Kruskal-Wallis test, *P *< 0.001), illustrating the generation of distinct cell types by the Neurog2(+) progenitors. Cells in the MCL were the largest, with an average diameter of 15.7 ± 0.4 μm (64 cells in 3 animals), while cells in the EPL had an average diameter of 13.4 ± 0.4 μm (57 cells in 3 animals) and cells in the GL 11.3 ± 0.5 μm (33 cells in 3 animals). The large GFP(+) cells in the MCL and EPL projected an apical dendrite to form a dendritic tuft in the GL. Secondary dendrites were present in the EPL running parallel to the MCL and axonal projection could often be seen leading towards the interior of the OB, presumed to be projecting out of the bulb, to cortical areas. These anatomical features define mitral cells as well as internal, middle and superficial tufted cells, the two populations of glutamatergic projection neurons of the OB.

The distribution of GFP(+) cells was strikingly different when animals were induced for recombination at later time points. In E17.5-injected animals (2,233 GFP(+) cells analyzed in 4 animals), only 3.1 ± 0.6 and 2.3 ± 0.8% of the GFP(+) cells could be seen in the MCL and EPL, respectively (Figure 2C,D). Instead, most GFP(+) cells were located in the GL (89.4 ± 5.7%). Most of these cells showed a dendritic tuft extending in a single glomerulus. Similarly, P0-injected animals (2,011 GFP(+) cells analyzed in 3 animals) showed a low number of cells in the MCL (9.3 ± 2.8%) and in the EPL (2.9 ± 0.6%) (Figure 2E,F). The vast majority of GFP(+) cells were again observed in the GL (82.5 ± 5.4%). Interestingly, the few cells observed in the MCL showed morphology different from mitral cells, with significantly smaller cell body diameter and poorly ramified dendrites (data not shown), suggesting the existence of a small population of Neurog2-derived interneurons in these deeper OB layers.

Together, these findings show that Neurog2(+) progenitors contribute to the sequential generation of mitral and tufted cells at early developmental stages, but also to a large population of smaller diameter, predominantly juxtaglomerular neurons at later developmental stages.

### Expression of Tbr1, Tbr2 and HuC/D by Neurog2-derived juxtaglomerular neurons

We went on to investigate the large population of juxtaglomerular neurons generated by Neurog2(+) progenitors. The expression of neuronal subtype markers by Neurog2(+) progenitors was assessed at P21, following tamoxifen injections at the same three time points mentioned above.

We first assessed the expression of the neuronal markers NeuN and HuC/D by Neurog2-derived neurons. These two neuronal markers label two largely non-overlapping populations of neurons in the adult OB (Additional file 3). Independently of their birthdates, the juxtaglomerular Neurog2-derived neurons showed a consistent lack of NeuN expression (0.18%, 2 of 1,135 GFP(+) cells analyzed). In clear contrast, GFP(+) cells were frequently seen to be HuC/D positive (94.5%, 153 of 162 GFP(+) cells investigated).

**Figure 3:**
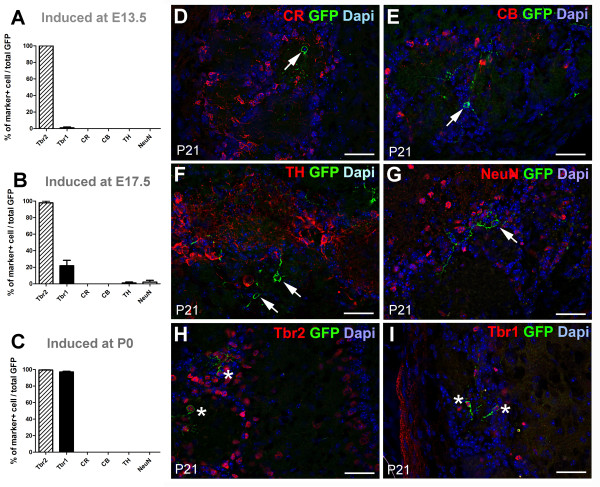
**Ngn2 progenitors sequentially generate Tbr2- and Tbr1-expressing neurons during embryonic development**. **(A-C) **Antigenic properties of Neurog2-derived OB juxtaglomerular neurons of P21 *Ngn2CreERT2 *× TaukiGFP mice. The recombination was induced at E13.5 (A), E17.5 (C) and at birth (E) by a single injection of tamoxifen. Error bars indicate S.E.M. **(D-I) **High field images of the pattern of expression of the markers used to identify subpopulations of juxtaglomerular OB neurons. All images were acquired from animals induced at birth and sacrificed at P21. Arrows point to cells negative for the markers tested. Asterisks indicate cells positive for Tbr2 and Tbr1. Scale bar: 50 μm in all panels. CB, calbindin; CR, calretinin; NeuN, neuronal nuclear antigen; Tbr1 and Tbr2, T-box protein 1 and 2; TH, tyrosine hydroxylase.

We next checked for expression by Neurog2-derived GFP(+) cells of the calcium binding proteins calretinin (CR) and calbindin (CB) as well as the dopamine synthesis enzyme tyrosine hydroxylase (TH), three markers delineating distinct classes of GABAergic interneurons in the GL. No expression of CR and CB could be observed in Neurog2-derived neurons at any of the time points investigated (1,101 and 1,040 cells, respectively; Figure 3). Only two cells were seen to be positive for TH in one of the E17.5-injected animals (0.17%, 2 of 1,195 GFP(+) cells analyzed). These data show that Neurog2-derived neurons do not express markers of periglomerular (PG) interneurons. Finally, we assessed the expression of the transcription factors Tbr1 and Tbr2 by the GFP(+) cells. These markers are known to label mitral and tufted cells as well as some juxtaglomerular cells [23,24]. In agreement with these previous findings, Tbr1 and Tbr2 immunostaining revealed a high number of positive cells in the MCL, EPL and GL (Additional file 4). Despite showing a similar distribution, we found that the staining for Tbr2 was stronger and more widespread than that of Tbr1 in the GL and EPL as revealed by counting the number of DAPI(+) cells expressing the two markers throughout the OB layers (Additional file 4).

Interestingly, the expression of these two markers significantly differs in juxtaglomerular cells generated at early and late developmental time points (Figure 3). Thus, while Tbr2 stably labeled most of the Neurog2-derived neurons at the three time points investigated (100% at E13.5, 97.9 ± 2.2% at E17.5 and 97.1 ± 0.7% at P0; 1,158 cells analyzed in 10 animals), Tbr1 predominantly labeled late-born Neurog2-derived neurons (unpaired *t*-test, *P *< 0.0001). In E13.5 Tam-injected animals, only 1 ± 0.96% of GFP(+) cells expressed Tbr1 (6 of 347 cells investigated in 3 animals). This percentage increased to 21.9 ± 6.5% in E17.5 Tam-injected animals (108 GFP(+) cells analyzed in 3 animals), with the highest proportion of co-labeling seen in P0 Tam-injected animals, with 97.1 ± 0.7% of the Neurog2-derived neurons expressing Tbr1 (368 GFP(+) cells analyzed in 3 animals).

These results indicate that Neurog2-derived progeny gradually populate the GL throughout the later stages of embryogenesis, generating first a population of Tbr2-positive juxtaglomerular cells (defined from here on as Tbr1(-)), followed by a second population of cells co-expressing Tbr1 and Tbr2 (defined from here on as Tbr1(+)).

### Glutamatergic nature of olfactory bulb Tbr1- and Tbr2-positive cells

**Figure 4:**
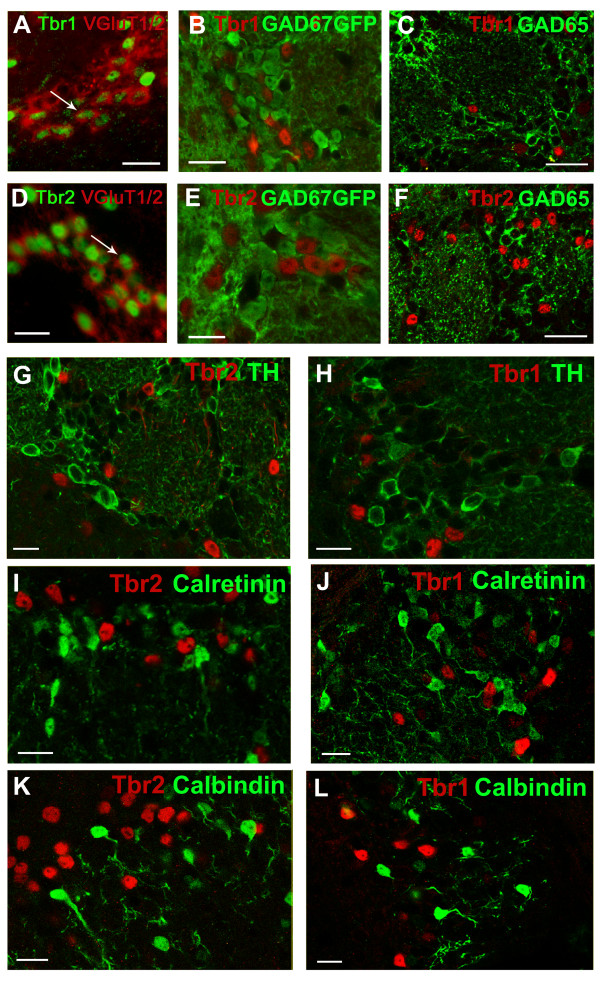
**Glutamatergic phenotype of Tbr2- and Tbr1-positive juxtaglomerular OB neurons**. **(A,D) **Tbr2 and Tbr1 are found in VGluT-positive cells, as revealed by combining *in situ *hybridization for VGluT1/2 (here imported into the red channel) and immunostaining in the green channel. Images are taken from the GL. **(B,C,E,F) **Tbr2 and Tbr1 do not co-localize with GAD67-GFP (B,E) nor GAD65 (C,F), confirming the non-GABAergic identity of these cell populations. Images are from the GL. **(G-L) **Tbr2 and Tbr1 do not co-localize with known periglomerular cell markers (TH, CR, CB or parvalbumin) in the GCL. Scale bars: 50 μm in (A-D); 20 μm in (G-R).

In order to confirm the glutamatergic nature of Tbr1(+) and Tbr2(+) neurons, we first tested whether these cells expressed vesicular glutamate transporters (VGluTs). Given the ubiquitous nature of the amino acid glutamate, *in situ *hybridization for VGluTs has come to be recognized as the most reliable method to label the cell bodies of glutamatergic neurons [25]. In agreement with recent findings in the rat OB [26], we found that, in the mouse OB, VGluT1 and 2 showed marked differences in their distributions through the OB layers. A larger proportion of VGluT1-positive cells was observed in the mitral cell layer, as well as strong expression at the boundary of the GL and EPL. By contrast, in addition to VGluT2 expression in the MCL and EPL, a large proportion of VGluT2-positive cells was found in the GL, many found more peripherally than the VGluT1(+) expression (Additional file 5). Whilst the majority of VGluT1/2(+) cells were Tbr2(+), only a smaller fraction was positive for Tbr1, confirming the smaller size of this second cell population (Figure 4A,D).

To further confirm the glutamatergic nature of Tbr1- and Tbr2-labeled juxtaglomerular cells, we next assessed their expression of GABAergic markers. We took advantage of the availability of knock-in GAD67-GFP mice [27]. We found no co-localization of Tbr1 and Tbr2 with GAD67-GFP (853 Tbr1 and 2,707 Tbr2 cells analyzed, respectively, in 3 animals; Figure 4B,E). The second isoform of glutamic acid decarboxylase (GAD65) was also absent in Tbr1- and Tbr2-positive cells, as visualized using an antibody specific for the GAD65 isoform [28]. Again, we found no overlap of Tbr1 or Tbr2 with GAD65 (135 Tbr1 and 978 Tbr2 cells analyzed, respectively, in 3 animals; Figure 4C,F), strongly suggesting an exclusive glutamatergic nature of Tbr1- and Tbr2-positive juxtaglomerular cells. Finally, we assessed the co-localization of the GABAergic markers CR, CB and parvalbumin, and the dopaminergic neuron marker TH. There has been much debate about the possible non-GABAergic nature of some CB and CR PG cells due to an absence of GABA, GAD67 and GAD65 immunodetection in these cells [29-31], although this seems to be predominantly due to efficiency of staining techniques [32]. Interestingly, we found no co-expression of Tbr2 or Tbr1 with parvalbumin (not shown) or CR, CB, and TH (Figure 4G-L; total of 397, 1,223, 1,663 and 898 cells counted, respectively).

In summary, our data provide evidence that Tbr1 and Tbr2 are reliable markers of developmentally generated, Neurog2-derived glutamatergic neurons throughout OB layers, in contrast to adult born glutamatergic neurons that do not express these two markers [10]. Moreover, our results suggest that whereas most Neurog2-derived glutamatergic neurons generated during development express Tbr2, a smaller population also expresses Tbr1, which might therefore represent a marker of a subpopulation of juxtaglomerular glutamatergic neurons.

### Differential expression of Tbr1 by external tufted interneurons and short axon cells in the glomerular layer

We next investigated further the nature of Tbr1(+) neurons by performing morphometric analysis of individually reconstructed juxtaglomerular neurons. Two populations of juxtaglomerular cells have been proposed to be glutamatergic: the external tufted cells and a subpopulation of short axon cells. These two cell populations can be classified according to their morphology [33]. External tufted cells most often show a single apical dendrite that ramifies into a single glomerulus while short-axon cells show multiple, sparsely branched dendrites that project predominantly in the GL [33].

**Figure 5:**
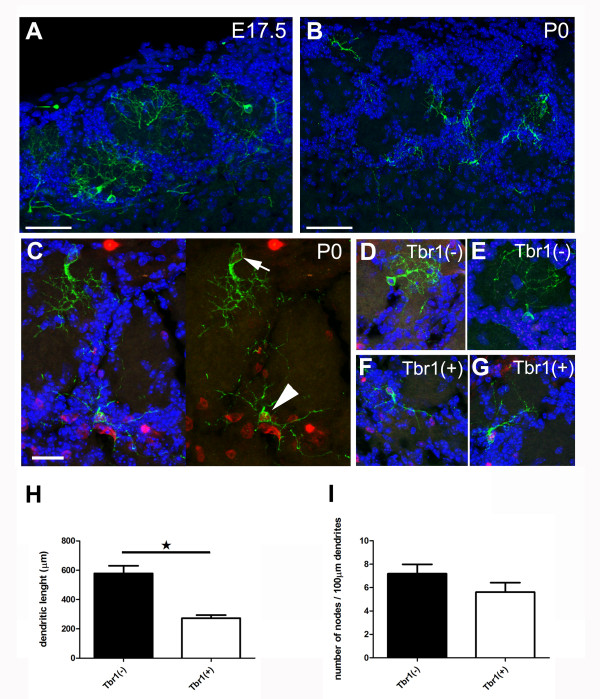
**Neurog2 progenitors generate juxtaglomerular neurons with distinct morphologies at E17.5 and P0**. **(A,B) **Maximal intensity projection of Neurog2-derived juxtaglomeular neurons generated at E17.5 (A) and P0 (B). Note the distinct morphology of the neurons generated at the two time points. While most neurons generated at earlier embryonic time points present intraglomerular dendritic arborization, those generated at birth show periglomerular dendritic arborization. **(C) **Maximal intensity projection of Tbr1-positive (arrowhead) and Tbr1-negative (arrow) neurons. **(D-G) **Other illustrative examples of maximal intensity projection showing the dendritic arborization of Tbr1-negative (D,E) and Tbr1-positive (F-G) neurons. **(H,I) **Tbr1 immunoreactivity identifies juxtaglomerular neurons with distinct morphologies as illustrated by the smaller dendritic arbor (H), while no differences in their branching ratio could be observed (I). Scale bars: 50 μm in (A-C); 10 μm in (C-F). Error bars indicate S.E.M.; *P < 0.05, determined by unpaired t-test.

**Figure 6:**
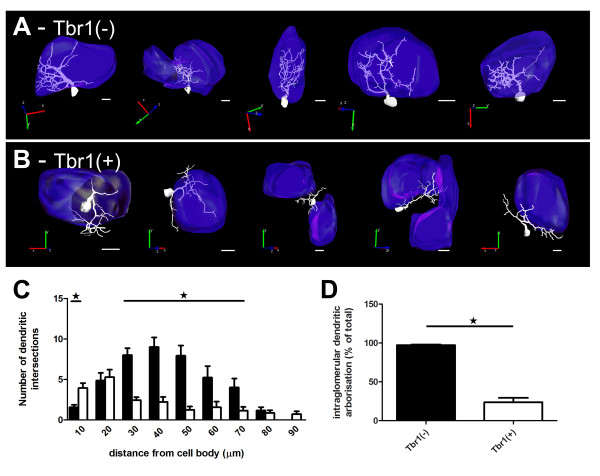
**Tbr1 immunoreactivity distinguishes short axon cells from surrounding tufted juxtaglomerular interneurons**. **(A,B) **Neurolucida reconstruction of individual Neurog2-derived juxtaglomerular neurons and of the associated glomeruli that are negative (A) or positive (B) for Tbr1. A three-dimensional drawing of individual glomeruli is shown in blue. **(C) **Sholl analysis of the two populations of cells reveals a tufted arborization of the Tbr1-negative population of juxtaglomerular cells. **(D) **Quantification of the percentage of intrabulbar dendritic arborization. Note the very pronounced differences observed between the two cell populations, with Tbr1-negative juxtaglomerular neurons showing intraglomerular extending dendrites while Tbr1-positive neurons show preferential periglomerular dendrites. Scale bars: 20 μm in (A); 15 μm in (B-E); 10 μm in (F,G). Error bars indicate S.E.M.; *P < 0.05, determined by unpaired t-test.

Beside their antigenic heterogeneity, the morphology of the GFP(+) cells born at E17.5 and P0 appeared to be very different, with most cells showing dendrites filling up nearby glomeruli at the earlier time point, whereas, a large number of the P0-generated cells showed clear PG dendritic arborization (Figure 5A,B). These pronounced differences in dendritic arborizations were clearly visible between Neurog2-derived Tbr1(+) and Tbr1(-) juxtaglomerular neurons born at P0 (Figure 5C-G). The dendritic arborization of randomly selected GFP(+) cells was reconstructed by performing neurolucida drawing from confocal stacks (14 Tbr1(+) cells and 16 Tbr1(-) cells). Tbr1(+) cells showed small dendritic arbors when compared to the Tbr1(-) cells (272.9 ± 20.4 μm compared to 569.2 ± 45.7 μm; unpaired t-test, *P *< 0.01; Figure 5H), reflecting the more compact dendritic arborization of Tbr1(-) cells that could more readily be observed on a single tissue section. The branching ratio was not, however, significantly different between Tbr1(+) and Tbr1(-) cells (7.2 ± 0.8 versus 5.6 ± 0.8; unpaired *t*-test, *P *> 0.05; Figure 5I). Sholl analysis of the two populations of cells also underlined major morphological differences, with Tbr1(-) cells showing a unipolar tufted morphology while the majority of Tbr1(+) cells showed multipolar morphologies, as reflected by the higher number of intersections close to the cell body. More distally, the Tbr1(+) cells showed fewer ramifications, resulting in a smaller and more stable number of intersections at increasing distances (Figure 6C). Finally, there were clear differences in the intraglomerular versus PG location of the dendritic arborizations of Tbr1(+) and Tbr1(-) cells. Thus, while most Tbr1(-) cells showed clear intraglomerular dendritic arborization, with 97.4% of the dendrites branching inside a single glomerulus (Figure 6A,D), Tbr1(+) cells showed primarily a PG dendritic arborization, with only 23.8% of their dendrites penetrating glomeruli (Figure 6B,D).

All together, these anatomical features identify Tbr1(+) cells as short axon cells, while Tbr1(-) cells are external tufted cells. These two populations of glutamatergic neurons derive from Neurog2-expressing progenitors and are generated at different embryonic stages, with the birth of external tufted cells preceding that of short axon cells.

### Extrabulbar origin of some Neurog2-derived juxtaglomerular cells at perinatal ages

**Figure 7:**
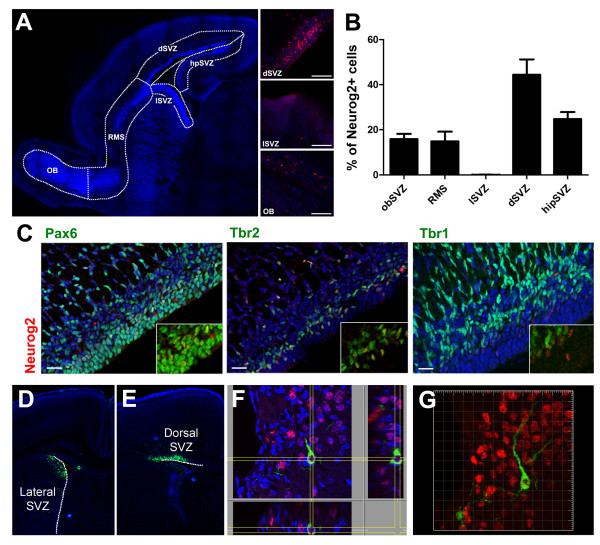
**Some glutamatergic periglomerular interneurons are of extrabulbar origin at perinatal ages**. **(A,B) **Distribution of Neurog2 protein throughout the SVZ, RMS and OB. (A) The P0 forebrain SVZ divided into five areas: olfactory bulb SVZ (obSVZ); RMS, also containing the more rostral portion of the lateral ventricle; lateral SVZ (lSVZ); dorsal SVZ (dSVZ, also named subcallosal SVZ); and hippocampal SVZ (hpSVZ). (B) The percentage of Neurog2-positive cells found in each area in a 40-μm thick sagital section from three independent animals. Error bars indicate S.E.M. **(C) **Co-staining of Neurog2 protein with Pax6, Tbr2 and Tbr1 showing a similar sequential expression of the four markers as observed at earlier developmental stages in the OB. Neurog2 protein shows a strong co-localization with Pax6 and Tbr2, but is down-regulated before Tbr1 expression. **(D-G) **GFP(+) cells arising from the dorsal SVZ give rise to some Tbr2-expressing juxtaglomerular OB neurons. Electroporation at birth of a GFP plasmid in the lateral (D) and dorsal (E) walls of the lateral ventricles at birth. **(F,G) **Only the dorsal electroporated animals show some Tbr2-positive juxtaglomerular neurons 12 days later, illustrating the extrabulbar origin of some OB glutamatergic juxtaglomerular neurons. Scale bars: 100 μm in (A, inserts); 20 μm in (C).

We went on to assess the origin of the late generated Neurog2-derived neurons. As shown in Figure 1, the pattern of Neurog2 expression changes significantly over time, with Neurog2-positive cells being visible in more caudal ventricular regions at E17.5 and P0. Immunodetection of Neurog2 revealed that a significant proportion of the cells expressing Neurog2 at this time are found in or right below the dense layer of DAPI-positive cells lining the lateral ventricle (Figure 7A,B), suggesting that at least some cells born at this late time point arise from the lateral ventricle and migrate through the RMS to the OB region. Interestingly, while Neurog2(+) cells could be observed in the dorsal and hippocampal subregions of the lateral ventricles, no cells could be observed in the lateral walls.

In the dorsal aspect of the lateral ventricles, Neurog2(+) progenitors show the same transcriptional profile observed in the OB ventricle at E13.5, with most Neurog2(+) cells co-expressing Tbr2 (Figure 7C). In order to confirm the contribution of the dorsal SVZ to OB interneurons perinatally, GFP plasmids were electroporated into the dorsal or lateral SVZ of P1 mice (Figure 7D,E). Shortly after electroporation (that is, 48 hours), GFP(+) cells were only observed in the dorsal (Additional file 6) or lateral walls of the lateral ventricle. No GFP(+) cells were observed at this early time point in the RMS or OB, excluding the possibility of direct labeling of intrabulbar progenitors (Additional file 6). At 12 days post-electroporation, many GFP-expressing cells could be detected throughout the OB. In the dorsally electroporated animals (n = 4), out of 342 GFP(+) cells seen in the GL, we identified 5 that unmistakably co-localized with Tbr2 (Figure 7F,G). However, no co-localization with Tbr2 was found in the laterally electroporated animals (n = 3; 241 GFP(+) cells analyzed).

These results indicate that cells born in the dorsal region of the perinatal SVZ migrate to the OB and mature to form glutamatergic neurons. A small population of OB glutamatergic neurons therefore has an extrabulbar origin during OB development.

## Discussion

OB development, in a similar manner to cortical development, has traditionally been thought to occur in two phases. The first stage concerns the production of glutamatergic projection neurons, which are born in the VZ and migrate radially into the OB. In the second stage, GABAergic interneurons arrive from extrabulbar origins (that is, first from the lateral ganglionic eminence and then from the postnatal RMS and lateral ventricle SVZ). In this study we show that there is considerable overlap between these two stages, with a significant number of glutamatergic OB neurons continuing to be born up to perinatal ages. Our results show the existence of a large population of intrabulbar Neurog2(+) progenitors at early developmental stages, when OB projection neurons are produced. At later stages, juxtaglomerular glutamatergic interneurons - that is, external tufted cells and short axon cells - are sequentially produced by progenitors from both intrabulbar and extrabulbar origins.

The decision to differentiate into a glutamatergic versus GABAergic neuronal phenotype is a binary fate choice in the developing telencephalon. Forebrain glutamatergic neurons have a pallial origin while GABAergic neurons are of subpallial origin. Several studies have demonstrated the central role of the proneural bHLH proteins Neurog2 and Mash1 in specifying these two classes of neurons [14,34,35]. In agreement with these studies, our fate mapping study confirms the exclusive glutamatergic fate of Neurog2-expressing progenitors in the developing OB. Our immunostaining experiments reveal that, at all time points investigated, Neurog2 is part of a transcriptional cascade comprising Pax6, Tbr2 and Tbr1. This transcriptional cascade, previously observed in the developing neocortex [11] and in the adult dentate gyrus [36], has been proposed to represent a generic program in the specification of glutamatergic neurons [12]. As illustrated here, however, the glutamatergic neuron subtypes produced at different developmental stages differ greatly in their morphology, pattern of projection and possibly electrophysiological properties. Thus, while large projection neurons are produced at early embryonic stages, smaller juxtaglomerular neurons are produced at later stages. These juxtaglomerular neurons show marked differences in their morphologies, with the dendritic arborization of late-born neurons becoming restricted to the GL. The nature of the signals determining the acquisition of these distinct cellular fates remains unknown. As in the neocortex, it is likely that diverse transcription factors act in concert with the generic glutamatergic transcriptional program discussed above to generate this diversity [37]. Alternatively, external constraints, such as the gradual development of the dense innervation in the glomeruli by neurons from the olfactory epithelium, may prevent dendritic growth inside glomeruli at late developmental stages. Interestingly, such PG dendritic arborizations are observed in all postnatal and adult-born subclasses (that is, GABAergic and glutamatergic) of adult-born OB interneurons [10,38].

Our results show that none of the Neurog2-derived juxtaglomerular neurons express classical markers of GABAergic PG neurons (that is, CR, CB, TH and parvalbumin). Instead, we show that all Neurog2-derived cells are positive for the T-box protein Tbr2, while some also express Tbr1. These two proteins represent reliable markers of developmentally born OB glutamatergic neurons as demonstrated by *in situ *hybridization for VGlut1 and 2, and confirmed by the lack of co-expression with the GABAergic markers GAD67 and GAD65. The absence of CR(+) PG neurons in the Neurog2 progeny is of particular interest. There has been much debate about the possible non-GABAergic nature of a large fraction of CR(+) PG neurons due to an absence of GABA, GAD67 and GAD65 immunodetection in these cells [29-31], although this seems to be predominantly due to efficiency of staining techniques [32]. Our results suggest that the vast majority of VGlut1/2 positive cells are Tbr1/2 positive, and argue against the existence of a large number of CR(+) glutamatergic neurons.

The absence of TH(+) PG neurons in the Neurog2 progeny is also of significance. Previous work has shown a role for Neurog2 in the generation of dopaminergic neurons in the midbrain [39,40]. In contrast, our fate mapping analysis shows only two Neurog2-derived PG neurons expressing the dopaminergic marker TH (representing <0.2% of the Neurog2 progeny) in the developing OB. Interestingly, previous studies have shown an involvement of Pax6 in the generation of PG dopaminergic neurons during development as well as in adulthood [41-44]. Considering the significant degree of overlap of Pax6 and Neurog2 in the developing forebrain, as well as in the dorsal aspect of the lateral ventricle at later stages, it might appear surprising that more TH(+) neurons were not observed in the Neurog2 progeny. Thus, during development, TH(+) OB interneurons might originate exclusively from the dorsal lateral ganglionic eminence where Pax6 expression overlaps with Mash1, but where Neurog2 expression is absent [45,46]. Alternatively, it is possible that several populations of pallial progenitors expressing different levels of Neurog2 co-exist, with our Cre-lox approach preferentially targeting cells expressing high levels of Neurog2. Pax6 is known to regulate Neurog2 directly, in a concentration-dependent manner [47], making the existence of a Pax6/Neurog2^HIGH ^and a Pax6/Neurog2^LOW ^population of pallial progenitors conceivable. In such a model, the level of expression of Neurog2 might represent a switch in GABAergic/glutamatergic fate acquisition of OB juxtaglomerular neurons, and explain the involvement of pallial progenitors expressing Emx1 in OB GABAergic neurogenesis [48]. A detailed analysis of the *Neurog*^GFP/GFP ^mouse (that is, Neurog2 knock-out) will be necessary to further explore this interesting possibility.

Similarly to findings challenging the exclusive extrabulbar origin of OB GABAergic interneurons [3], our findings also challenge the idea of an exclusive intrabulbar origin of glutamatergic OB neurons. Both the pattern of GFP expression in the forebrain of *Neurog2*^+/GFP ^and the quantification of Neurog2-expressing cells in neonatal animals illustrate the widespread distribution of these cells from the OB to more caudal areas (that are, RMS and lateral ventricles), which contribute to OB neurogenesis at late developmental stages. To confirm the extrabulbar origin of some Neurog2-derived neurons, we used electroporation of GFP plasmids into radial astrocytes of the lateral ventricle of newborn mice [49]. We modified this technique to specifically target subregions of the lateral ventricle (MF and OR, manuscript in press). In agreement with the exclusive dorsal location of Neurog2(+) cells, only the dorsal electroporation resulted in the successful labeling of OB neurons expressing Tbr2 after 12 days, confirming the extrabulbar origin of some of these neurons. The exclusion of GABAergic markers and the expression of VGlut1/2 by Tbr2(+) cells in the adult animal support the glutamatergic nature of these neurons. It is to be noted that the number of Tbr2 juxtaglomerular cells found originating from the lateral ventricle was low, but this number was probably an underestimation. First, the electroporation was very local, with only a fraction of the radial glia being electroporated. Second, a large number of Neurog2(+) progenitors can be seen at P0 in the RMS, a population of progenitors that was not targeted by the electroporation. Finally, some Neurog2-derived glutamatergic neurons generated at birth might not express Tbr1/2, as previously shown for most glutamatergic neurons generated in 2-month-old mice [10]. Whilst our results clearly show the extrabulbar origin of a population of glutamatergic juxtaglomerular cells, the above approaches do not yet allow us to determine the size of this population.

Of particular interest is the differential expression of Tbr1 in the two populations of juxtaglomerular glutamatergic interneurons. Based on morphological criteria, our results suggest that Tbr2 is a pan-glutamatergic marker for embryonic generated OB neurons, while it does not label adult generated glutamatergic OB interneurons [10], and Tbr1 preferentially labels short axon cells and is excluded from external tufted cells.The term 'external tufted cell' has recently been applied only to tufted cells found in the GL, while those in the EPL are referred to as superficial, middle or internal tufted cells, dependent on the position of the cell body [50]. External tufted cells, whilst they have an intraglomerular dendritic tuft that appears very similar to that of other tufted cells, are not thought to project out of the OB, but rather to other areas of the GL [20,51]. Superficial short-axon cells by contrast have no dendritic tuft but are long range interneurons, projecting sparsely branching dendrites through the GL to contact distant glomeruli [33]. Our three-dimensional reconstruction of single Tbr1(-) and Tbr1(+) cells supports the classification of these cells in these two categories. Importantly, the lack of TH expression by Tbr1(+) cells distinguishes them from other interglomerular GABAergic neurons recently described in the mouse GL [52,53]. The expression of Tbr2 in most OB glutamatergic neurons contrasts with the cortex, where only Tbr1 expression is observed in mature excitatory neurons, while Tbr2 is absent [23]. The reasons for this molecular, area-specific heterogeneity, as well as the function of the expression of these transcription factors in populations of mature glutamatergic neurons, are at present unknown.

## Conclusions

**Figure 8:**
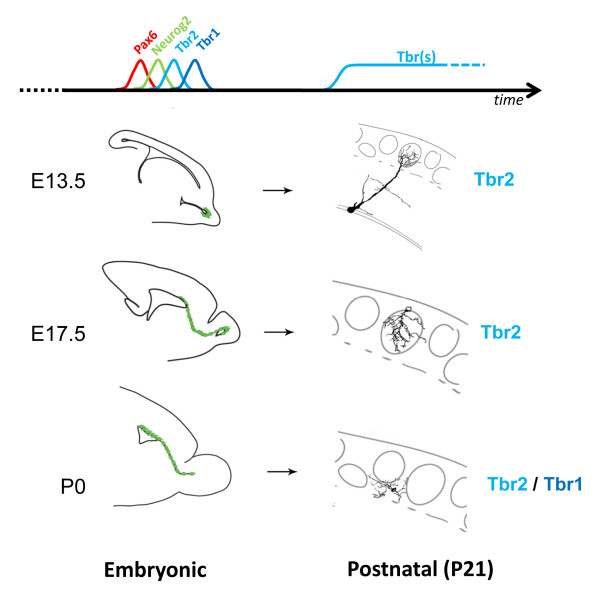
**Schematic representation of results obtained in this study**. The left-hand drawings represent the pattern of Neurog2 expression in the mouse OB, RMS and dorsal wall of the lateral ventricle at the developmental stages analyzed in this study (E13.5, E17.5 and P0). Note that at all stages progenitors sequentially and transiently express Pax6, Neurog2, Tbr2 and Tbr1. The right drawings are neurolucida reconstructions of mature neurons produced at these different developmental stages. Note the different morphologies. All neurons express Tbr2 independently of their date of birth, but only neurons generated at late developmental stages also express Tbr1.

Our results demonstrate the sequential generation by Neurog2(+) progenitors of distinct populations of OB glutamatergic neurons (Figure 8). The description of this developmental timeframe together with the use of the *Neurog2iCreERT2 *mice will allow future studies aimed at studying the physiology of these glutamatergic neuron subtypes, in particular of juxtaglomeurular neurons, whose exact role in olfactory processing remains to be explored [17,18].

## Materials and methods

### Animals

Electroporation experiments were performed in agreement with the Canton of Zurich veterinary office guidelines. All other experiments were done in agreement with the United Kingdom Animals Act 1986 for scientific procedures.

Heterozygous *Neurog2*^+/GFP ^mice with an enhanced GFP cassette knocked-in the *Neurogenin-2 *locus [54]. *Neurog2iCreERT2 *mice were generated using a BAC encoding *iCreERT2 *under the control of the *Neurog2 *promoter. The BAC was injected into blastocysts that were implanted in pseudo-pregnant females. The resulting mice were tested by PCR for the presence of the transgene. When crossed with the appropriate reporter line (RosaYFP or TauGFP) and induced with tamoxifen, the reporter is expressed in a pattern that is consistent with Neurog2 endogenous expression in the mouse brain. RosaYFP mice are homozygous reporter mice carrying the yellow fluorescent protein (*YFP*) transgene inserted in the Rosa26 locus [55]. TauGFP mice are homozygous Cre-reporter mice carrying the *GFP *transgene inserted in the neuron-specific Tau locus [22]; in this mouse, a membrane bound-GFP, consisting of a fusion of GFP with a plasmalemmal targeting sequence of the protein MARCKS [56], was expressed upon recombination. This allowed the visualization of the entire morphology of the recombinated cells.

Genotyping of all transgenic strains was performed by PCR. Genotyping of the *Neurog2GFP *mice used 5'-3' primer GGA CAT TCC CGG ACA CAC A C and 3'-5' primer GCA TCA CCT TCA CCC TCT CC with 35 cycles of 94°C/1 minute, 60°C/1 minute and 72°C/1 minute to detect the knock-in form, and 5'-3' primer GAC ATT CCC GGA CAC ACA C and 3'-5' primer GAG CGC CCA GAT GTA ATT GT with 35 cycles of 94°C/30 s, 62°C/1 minute and 72°C/1 minute were used to detect the wild-type *Neurog2 *gene. To detect the knock-in of Cre recombinase in the *Neurog2iCreERT2 *mice, 5'-3' primer AGA TGC CAG GAC ATC AGG AAC CTG and 3'-5' primer ATC AGC CAC ACC AGA CAC AGA GAT were used with 35 cycles of 94°C/30 s, 62°C/1 minute and 72°C/1 minute. Genotyping of the TauGFP reporter mice used 5'-3' primer AAG TTC ATC TGC ACC ACC G and 3'-5' primer TCC TTG AAG AAG ATG GTG CG with 35 cycles of 95°C/30 s, 60°C/30 s, and 72°C/1 minute.

### Tamoxifen injections

Pregnant *Neurog2iCreERT2 *females were injected intraperitoneally with 6 mg of 4-hydroxytamoxifen (4-OHT) either on E13.5 or E17.5. Alternatively, P0 pups were injected with 0.5 mg tamoxifen subcutaneously. The 4-OHT solution was prepared by adding 1 ml of absolute ethanol to 50 mg of 4-OHT powder (Sigma no. H6278, St. Louis, Missouri, USA). The powder was put in a suspension by manual pipetting. Corn oil (4 ml; Sigma no C8267) was added to the 4-OHT suspension, and the mix was incubated at 37°C for approximately 90 minutes and agitated a few times. If needed, the mix was shortly incubated at 65°C to dissolve all the 4-OHT. Tamoxifen solution was prepared by adding 1 ml ethanol to 200 mg of tamoxifen. This solution was then sonicated and when all the tamoxifen had dissolved, 9 ml of corn oil was added to obtain a 20 mg/ml solution. Tamoxifen-induced mice were perfused at age P21, 3 weeks after birth.

### Electroporation

P1 mice were electroporated with GFP plasmid as described in detail elsewhere [49]. The plasmids were purified using the Qiagen EndoFree Plasmid Maxi Kit (Qiagen, no. 12362, Valencia, California, USA) according to the manufacturer's protocol. DNA was resuspended in sterile phosphate-buffered saline at a concentration of 5 μg/μl, which was determined using the NanoDrop^® ^ND-1000 UV-Spectrophotometer (NanoDrop Technologies, Wilmington, Delaware, USA).

Animals were perfused at either 2 or 12 days post-electroporation. Electroporation was performed on P1 mice of the CD1 strain (Swiss mice) using a method adapted from [49]. Pups were anesthetized by hypothermia before being placed in a stereotaxic rig. Injections were performed at the midpoint of a virtual line connecting the eye with Lambda. Injections were made using a 34G Hamilton syringe at a depth of 2.5 mm. Correct injections were confirmed by visual inspection due to the filling of the lateral ventricle with the dark solution. Animals in which the injection had been successful were subjected to five electrical pulses (90 V, 50 ms, separated by 950-ms intervals) using the CUY21 edit device (Nepagene, Chiba, Japan) and 10-mm tweezer electrodes (CUY650P10; Nepagene) coated with conductive gel. Positioning of the electrodes on each side of the head caused transfer of the plasmids into the lateral SVZ, whereas positioning of the positive electrode more dorsally caused electroporation into the dorsal SVZ. Electroporated animals were warmed on a heat mat and returned to the mother.

### Collection of tissue

*Neurog2*^+/GFP ^embryos were collected at E13.5 or E17.5 following an overdose of anesthetic to the pregnant female. The age of the embryos was calculated from the morning that vaginal plugs were observed, which was considered to be E0.5. Embryos were collected in dissection solution, the outer membranes removed and the entire head then fixed by immersion in 4% paraformaldehyde for 4 days, followed by cryoprotection in 30% sucrose solution. P0 and adult brains were fixed by transcardial perfusion with 4% paraformaldehyde, followed by overnight post-fixation and cryoprotection with 30% sucrose solution. OB cryosections were obtained at 40 μm. A minimum of three animals were used for all quantifications.

### Immunohistochemistry

Sections were blocked and permeabilized with 5% normal horse serum and 0.4% Triton X-100 in phosphate buffer for 2 hours, followed by an overnight incubation with the primary antibody dissolved in the same blocking medium. After washing, the sections were incubated for 2 hours with the corresponding secondary antibody. DAPI counterstain was performed for all stainings. The complete list of antibodies used in this study can be found in Table 1 at the end of this manuscript

**Table 1:** Antibodies used in this study

Target protein	Antigen	Species	Dilution	Source	Antibody characterization
GFP	Protein isolated from *Aequorea victoria*	Rabbit	1:500	Invitrogen	Recognizes a single band at 27 kDa on western blot of GFP transgenic mouse brain. No immunostaining seen in sections not containing GFP
GFP	Recombinant full length protein	Chicken	1:1,000	Abcam ab13970	Recognizes a single band at 27 kDa on western blot of GFP transgenic mouse brain. No immunostaining seen in sections not containing GFP
Tbr2	Synthetic peptide from mouse Tbr2	Rabbit l	1:1,000	Abcam ab23345	The antibody recognizes a single band at 72 kDa on a western blot of whole embryonic mouse brain (manufacturer's website). No western blot staining after neutralization of antibody with Tbr2 peptide
Tbr1	Synthetic peptide from mouse Tbr1	Rabbit	1:1,000	Millipore AB9616	The antibody recognizes the 68 kDa Tbr1 protein on a western blot (manufacturer's data sheet). No staining seen in Tbr1 knock-out animal (manufacturer's personal communication)
Neurog2	Carboxyl terminus peptide of Neurogenin 2 of human origin	Goat	1:100	Santa Cruz SC19233	No western blot staining after neutralization of antibody with Neurog2 peptide
Calretinin	Recombinant human calretinin-22k	Mouse	1:1,500	Swant 6B3	This antibody specifically recognizes the 29-kDa band on immunoblots of brain extracts. The antibody staining is absent in calretinin knock-out mice (manufacturer's website)
Calbindin	recombinant rat calbindin D-28k	Rabbit	1:5,000	Swant CB38	This antibody specifically recognizes the 28-kDa band on immunoblots of brain extracts. The antibody staining is absent in calretinin knock-out mice (manufacturer's website)
Tyrosine hydroxylase	Tyrosine hydroxylase purified from PC12 cells	Mouse	1:250	Millipore MAB318	Recognizes a protein of approximately 59 to 61 kDa by western blot and does not cross-react with dopamine-hydroxylase, phenylalanine hydroxylase, tryptophan hydroxylase, or phenyl ethanolamine-N-methyl transferase on western blots (manufacturer's website). This antibody stained only cells with the typical morphology and positioning of dopaminergic periglomerular cells in the olfactory bulb
Parvalbumin	Parvalbumin purified from carp muscles	Mouse	1:5,000	Swant PV235	Specifically stains the 45Ca-binding spot of parvalbumin (MW 12,000 and IEF 4.9) in a two-dimensional 'immunoblot'. No antibody staining seen in a parvalbumin knock-out mouse (manufacturer's website).
Pax6	Peptide from the carboxyl terminus of the mouse Pax6 protein	Rabbit	1:250	Covance PRB-278P	The antibody recognizes a single band at 49 kDa on a western blot of whole embryonic mouse brain (manufacturer's personal communication)
NeuN	Purified cell nuclei from mouse brain	Mouse	1:500	Millipore MAB377	Recognizes two to three bands in the 46- to 48-kDa range and possibly another band at approximately 66 kDa. Given that NeuN is defined by the antibody, it cannot be characterized. However for further discussion see [58]
HuC/D	HuC/HuD neuronal protein	Mouse	1:250	Invitrogen A-21271	Recognizes the Elav family members HuC, HuD and Hel-N1, which are all neuronal proteins. Anti-HuC/D monoclonal 16A11 does not recognize HuR, another Elav family member that is present in all proliferating cells
GAD65	Affinity-purified GAD from rat brain	Mouse	1:1,000	Abcam AB26113	Recognizes selectively GAD65; extensively co-localizes with VGAT in GABAergic axon terminals [28]

Swant (Marly, Fribourg, Switzerland)

Abcam (Cambridge, County of Cambridgeshire, UK)

Millipore (Billerica, Massachusetts, USA)

Santa Cruz Biotechnology (Santa Cruz, California, USA)

Invitrogen (Carlsbad, California, USA)

Covance (Princeton, New Jersey, USA)

Double staining was performed by applying two primary and secondary antibodies simultaneously. However, for double stainings in which the primary antibody was produced in the same animal, a more complex protocol was needed [57]. Here we first applied the first primary antibody, and used a tyramide amplification kit from Invitrogen to amplify the fluorescent signal (Invitrogen, Carlsbad, California, USA). We then microwaved the sections for 5 minutes in 10 mM citric acid buffer, pH6, in order to denature the first primary antibody. The second primary and secondary could then be applied with no risk of cross-reaction. Appropriate controls were performed by omitting the primary antibody from the second immunoreaction. These control sections showed no signal, indicating that the secondary antibody against the second primary applied was not picking up any signal from the first primary antibody applied.

### Imaging

A Leica DM6000 fluorescent microscope with a 40X/1.25 NA oil objective was used for cell counting. Co-localization studies and confocal images were obtained using a Leica SPE confocal laser scanning microscope with 40X/1.25 NA oil objective. Both microscopes used the Leica LAS AF software (Leica, Germany). Co-localization studies were done either by counting by eye from Confocal images or using the semi-automatic Imaris program (Bitplan, Zurich, CH, Switzerland). Digital images were processed using Adobe Photoshop. Only the contrast and brightness of figures were adjusted when necessary in order to improve clarity of images.

### Cell reconstruction

Three-dimensional reconstruction of individual juxtaglomerular cells and of their associated glomeruli was achieved by using the image stack module of the neurolucida software (MBF Bioscience, Williston, Vermont, USA for illustration). Sections (50 μm) of the OB were immunostained for Tbr2 and Tbr1 as described above. Randomly selected cells were imaged under a confocal microscope (objective 40 ×, NA 1.25). Image stacks encompassing the entire section thickness were collected with a z-step of 0.3 μm and a minimal resolution of 1,024 × 1,024 pixels.

### *In situ *hybridization

mRNA *in situ *hybridizations were performed on tissue fixed as for immunohistochemistry. The sections were cut to a thickness of 30 μm on a cryostat and mounted directly onto Superfrost slides. Two different mRNA probes were used. The VGluT1 probe was made using RT-PCR to obtain a cDNA fragment from within the *VGluT1 *gene. The following primers were selective for a region from the 3' end of *VGluT1*, and also incorporated SP6 and T7 sequences: forward primer, TAATACGACTCACTATAGGGTTTCGGGATGGAAGCCACG; reverse primer, ATTTAGGTGACACTATAGACGTCCTCCATTTCACTTTCGTC. The *vGluT2 *plasmid was the kind gift of Q Ma (Harvard Medical School). The probe was then made by *in vitro *transcription using a digoxigenin labeling kit (Roche Applied Science, Rotkreuz, Switzerland). Hybridization was done in a standard hybridization buffer, overnight at 60°C and the sections were then washed in decreasing concentrations of SSC before incubation for 2 hours with alkaline phosphatase-conjugated anti-digoxigenin antibody Fab fragment (Roche) diluted 1:500 in maleic acid buffer (Roche Applied Science, Rotkreuz, Switzerland). To visualize the bound phosphatase, a mixture of 3.375 mg/ml NBT and 1.75 mg/ml BCIP was applied to the sections overnight, or as long as required for the reaction to develop Images were obtained using a Leica LEITZ DMRB microscope (Leica Microsystems, Heerbrugg, Switzerland) and LuciaG software (Laboratory Imaging, Praha, Czech Republic).

## Abbreviations

4-OHT: 4-hydroxytamoxifen; BAC: bacterial artificial chromosome; bHLH: basic helix-loop-helix; CB: calbindin; CR: calretinin; E: embryonic day; EPL: external plexiform layer; GFP: green fluorescent protein; GL: glomerular layer; MCL: mitral cell layer; OB: olfactory bulb; P: postnatal day; PG: periglomerular; RMS: rostral migratory stream; SVZ: subventricular zone; TH: tyrosine hydroxylase; VGluT: vesicular glutamate transporter; VZ: ventricular zone.

## Competing interests

The authors declare that they have no competing interests.

## Authors' contributions

EW, MLP, MEF, MSB and OR carried out the experimental work. FG and MLP generated the *Neurog2iCreERT2 *mouse. OR conceived of the study and wrote the manuscript. MG, FG, MEF and MSB participated in critical reading of the manuscript. All authors read and approved the final manuscript.

## Supplementary Material

Additional file 1 **Figure S1: The expression of *iCreERT2 *follows *Neurog2 *expression in the E14.5 mouse telencephalon**. **(A-D') ***In situ *hybridization showing the expression of *iCreERT2 *(A,B,C,D) and *Ngn2 *(A',B',C',D') at different antero-posterior levels of the E14.5 mouse telencephalon (from anterior (A,A') to posterior (D,D') telencephalon. **(E,F) **Immunodetection of Neurog2 (E, top panel) and iCreERT2 (E, middle panel) in the newborn lateral ventricle reveals co-expression of the two markers in the same cells (DAPI nuclear counterstain; E, bottom panel). Note that while some cells are positive for both Neurog2 and iCreERT2 (arrow), some have downregulated Neurog2 but still express iCreERT2 (arrowhead) due to its longer half-life. Surrounding cells are consistently negative for the two markers (asterisk). Quantifications were performed by densitometry analysis (F). Grey values for DAPI, Neurog2 and iCreERT2 were measured in randomly selected DAPI(+) cells in the dorsal and ventral walls of the lateral ventricles of three *Neurog2iCreERT2 *animals (n > 50 cells per animal). Note the clear detection of iCreERT2 in the Neurog2(+) cell fraction. Note that iCreERT2 can also be detected in some Neurog2(-) cells, presumably cells that have downregulated Neurog2 but still express iCreERT2 due to its longer half-life. Click here for file

Additional file 2 **Figure S2: Short-term fate mapping of the Neurog2 progeny in the *Neurog2iCreERT2 *mice**. *Neurog2iCreERT2 *mice were crossed with RosaYFP reporter mice. Animals were injected with tamoxifen at P0 and scarificed 24 hours later. **(A-D) **Recombined cells could be observed at the transition of the lateral ventricle with the RMS (A) (the doted box on the DAPI counterstained cross-section indicates the approximate location of the GFP(+) cells shown in (B-D)). GFP immunostaining showed co-localization with Tbr2 (B) (100%, n = 32) but not with Dlx2 (C, D) (0%, n = 15), confirming the faithful expression of the Cre recombinase and the efficient labeling of the Neurog2 cell lineage. Click here for file

Additional file 3 **Figure S3: Neuronal phenotype of Tbr1/Tbr2-positive cells**. **(A,B) **Sequential double immunostaining for NeuN (green) and HuC/D (red) reveals different degrees of co-localization (yellow) of the two markers throughout the OB layers. **(C) **Percentage of DAPI-positive cells expressing either NeuN or HuC/D in each OB layer. **(D-G) **Double immunostaining of Tbr2 or Tbr1 with either NeuN or HuC/D. NeuN is excluded from the Tbr1- and Tbr2-positive cells while HuC/D preferentially labels the two OB glutamatergic neuron populations. Scale bars: 100 μm in (A); 20 μm in (D-G). Click here for file

Additional file 4 **Figure S4: Distribution of Tbr1- and Tbr2-positive neurons in the mouse olfactory bulb**. **(A,B) **Overview showing the distribution pattern of Tbr2 and Tbr1 immunoreactivity in the adult OB. **(C-F) **Graphs showing the quantification of the distribution of Tbr2 and Tbr1 in the different OB cell layers. Click here for file

Additional file 5 **Figure S5: Distribution of VGlut1- and VGlut2-positive neurons in the mouse olfactory bulb**. **(A-D) **Distribution pattern of VGluT2 and VGlut1 in the adult OB. VGluT1 *in situ *hybridization shows a strong signal in the MCL and a weaker signal at the boundary of the EPL and GL (A). VGluT2 mRNA is found both in the MCL and throughout the GL (C). DAPI counterstaining (B, D) allows the visualization of the distinct OB layers. **(E-F) **Graphs showing the quantification of the distribution of VGlut1 and VGlut2 in the different OB cell layers. When we considered the VGluT-positive cells as a proportion of DAPI(+) cells, we found a similar distribution. VGluT1(+) cells accounted for around 14% of DAPI(+) cells in the MCL, EPL1, and EPL3, with a smaller proportion in EPL2 and GL1. VGluT2(+) cells were more widely distributed between the layers. In the GL as a whole, VGluT2 accounted for 15% of DAPI(+) cells, 19% in the MCL, and a lower proportion in the EPL. Click here for file

Additional file 6 **Figure S6: Local expression of GFP after dorsal electroporation of the lateral ventricle**. **(A-E) **Native GFP expression observed 48 hours after electroporation of a GFP expression plasmid in the dorsal wall of the lateral ventricle of a newborn mouse at different caudo-rostral levels (from caudal (A) to rostral (E, showing the OB)). DAPI was used as a nuclear counterstain (blue). Scale bar: 1 mm in (A-D); 500 μm in (E). Click here for file

## References

[B1] Hinds JW Autoradiographic study of histogenesis in the mouse olfactory bulb. I. Time of origin of neurons and neuroglia J Comp Neurol 1968 134 287 304 10.1002/cne.901340304 5721256

[B2] Bayer SA 3H-thymidine-radiographic studies of neurogenesis in the rat olfactory bulb Exp Brain Res 1983 50 329 340 10.1007/BF00239197 6641865

[B3] Vergano-Vera E Yusta-Boyo MJ de Castro F Bernad A de Pablo F Vicario-Abejon C Generation of GABAergic and dopaminergic interneurons from endogenous embryonic olfactory bulb precursor cells Development 2006 133 4367 4379 10.1242/dev.02601 17038521

[B4] Wichterle H Turnbull DH Nery S Fishell G Alvarez-Buylla A *In utero *fate mapping reveals distinct migratory pathways and fates of neurons born in the mammalian basal forebrain Development 2001 128 3759 3771 11585802 10.1242/dev.128.19.3759

[B5] Gritti A Bonfanti L Doetsch F Caille I Alvarez-Buylla A Lim DA Galli R Verdugo JM Herrera DG Vescovi AL Multipotent neural stem cells reside into the rostral extension and olfactory bulb of adult rodents J Neurosci 2002 22 437 445 11784788 10.1523/JNEUROSCI.22-02-00437.2002 PMC6758684

[B6] Doetsch F Petreanu L Caille I Garcia-Verdugo JM Alvarez-Buylla A EGF converts transit-amplifying neurogenic precursors in the adult brain into multipotent stem cells Neuron 2002 36 1021 1034 10.1016/S0896-6273(02)01133-9 12495619

[B7] Altman J Autoradiographic and histological studies of postnatal neurogenesis. IV. Cell proliferation and migration in the anterior forebrain, with special reference to persisting neurogenesis in the olfactory bulb J Comp Neurol 1969 137 433 457 10.1002/cne.901370404 5361244

[B8] Bayer SA Neuron production in the hippocampus and olfactory bulb of the adult rat brain: addition or replacement? Ann N Y Acad Sci 1985 457 163 172 10.1111/j.1749-6632.1985.tb20804.x 3868311

[B9] Batista-Brito R Close J Machold R Fishell G The distinct temporal origins of olfactory bulb interneuron subtypes J Neurosci 2008 28 3966 3975 10.1523/JNEUROSCI.5625-07.2008 18400896 PMC2505353

[B10] Brill MS Ninkovic J Winpenny E Hodge RD Ozen I Yang R Lepier A Gascon S Erdelyi F Szabo G Adult generation of glutamatergic olfactory bulb interneurons Nature Neuroscience 2009 12 1524 1533 10.1038/nn.2416 19881504 PMC2787799

[B11] Englund C Fink A Lau C Pham D Daza RA Bulfone A Kowalczyk T Hevner RF Pax6, Tbr2, and Tbr1 are expressed sequentially by radial glia, intermediate progenitor cells, and postmitotic neurons in developing neocortex J Neurosci 2005 25 247 251 10.1523/JNEUROSCI.2899-04.2005 15634788 PMC6725189

[B12] Hevner RF Hodge RD Daza RA Englund C Transcription factors in glutamatergic neurogenesis: conserved programs in neocortex, cerebellum, and adult hippocampus Neurosci Res 2006 55 223 233 10.1016/j.neures.2006.03.004 16621079

[B13] Hodge RD Kowalczyk TD Wolf SA Encinas JM Rippey C Enikolopov G Kempermann G Hevner RF Intermediate progenitors in adult hippocampal neurogenesis: Tbr2 expression and coordinate regulation of neuronal output J Neurosci 2008 28 3707 3717 10.1523/JNEUROSCI.4280-07.2008 18385329 PMC6671086

[B14] Fode C Ma Q Casarosa S Ang SL Anderson DJ Guillemot F A role for neural determination genes in specifying the dorsoventral identity of telencephalic neurons Genes Dev 2000 14 67 80 10640277 PMC316337

[B15] Britz O Mattar P Nguyen L Langevin LM Zimmer C Alam S Guillemot F Schuurmans C A role for proneural genes in the maturation of cortical progenitor cells Cereb Cortex 2006 16 Suppl 1 i138 151 10.1093/cercor/bhj168 16766700

[B16] Shepherd GM Synaptic organization of the mammalian olfactory bulb Physiol Rev 1972 52 864 917 4343762 10.1152/physrev.1972.52.4.864

[B17] Hayar A Karnup S Ennis M Shipley MT External tufted cells: a major excitatory element that coordinates glomerular activity J Neurosci 2004 24 6676 6685 10.1523/JNEUROSCI.1367-04.2004 15282270 PMC6729710

[B18] Aungst JL Heyward PM Puche AC Karnup SV Hayar A Szabo G Shipley MT Centre-surround inhibition among olfactory bulb glomeruli Nature 2003 426 623 629 10.1038/nature02185 14668854

[B19] Hayar A Karnup S Shipley MT Ennis M Olfactory bulb glomeruli: external tufted cells intrinsically burst at theta frequency and are entrained by patterned olfactory input J Neurosci 2004 24 1190 1199 10.1523/JNEUROSCI.4714-03.2004 14762137 PMC6793566

[B20] Schoenfeld TA Macrides F Topographic organization of connections between the main olfactory bulb and pars externa of the anterior olfactory nucleus in the hamster J Comp Neurol 1984 227 121 135 10.1002/cne.902270113 6470206

[B21] Ozen I Galichet C Watts C Parras C Guillemot F Raineteau O Proliferating neuronal progenitors in the postnatal hippocampus transiently express the proneural gene Ngn2 Eur J Neurosci 2007 25 2591 2603 10.1111/j.1460-9568.2007.05541.x 17466019

[B22] Hippenmeyer S Vrieseling E Sigrist M Portmann T Laengle C Ladle DR Arber S A developmental switch in the response of DRG neurons to ETS transcription factor signaling PLoS Biol 2005 3 e159 10.1371/journal.pbio.0030159 15836427 PMC1084331

[B23] Kimura N Nakashima K Ueno M Kiyama H Taga T A novel mammalian T-box-containing gene, Tbr2, expressed in mouse developing brain Brain Res Dev Brain Res 1999 115 183 193 10.1016/S0165-3806(99)00064-4 10407135

[B24] Allen ZJ Waclaw RR Colbert MC Campbell K Molecular identity of olfactory bulb interneurons: transcriptional codes of periglomerular neuron subtypes J Mol Histol 2007 38 517 525 10.1007/s10735-007-9115-4 17624499

[B25] Gabellec MM Panzanelli P Sassoe-Pognetto M Lledo PM Synapse-specific localization of vesicular glutamate transporters in the rat olfactory bulb Eur J Neurosci 2007 25 1373 1383 10.1111/j.1460-9568.2007.05400.x 17425564

[B26] Ohmomo H Ina A Yoshida S Shutoh F Ueda S Hisano S Postnatal changes in expression of vesicular glutamate transporters in the main olfactory bulb of the rat Neuroscience 2009 160 419 426 10.1016/j.neuroscience.2009.02.048 19264112

[B27] Tamamaki N Yanagawa Y Tomioka R Miyazaki J Obata K Kaneko T Green fluorescent protein expression and colocalization with calretinin, parvalbumin, and somatostatin in the GAD67-GFP knock-in mouse J Comp Neurol 2003 467 60 79 10.1002/cne.10905 14574680

[B28] Chang YC Gottlieb DI Characterization of the proteins purified with monoclonal antibodies to glutamic acid decarboxylase J Neurosci 1988 8 6 2123 30 3385490 10.1523/JNEUROSCI.08-06-02123.1988 PMC6569335

[B29] Parrish-Aungst S Shipley MT Erdelyi F Szabo G Puche AC Quantitative analysis of neuronal diversity in the mouse olfactory bulb J Comp Neurol 2007 501 825 836 10.1002/cne.21205 17311323

[B30] Kosaka K Kosaka T synaptic organization of the glomerulus in the main olfactory bulb: compartments of the glomerulus and heterogeneity of the periglomerular cells Anat Sci Int 2005 80 80 90 10.1111/j.1447-073x.2005.00092.x 15960313

[B31] Kosaka K Kosaka T Chemical properties of type 1 and type 2 periglomerular cells in the mouse olfactory bulb are different from those in the rat olfactory bulb Brain Res 2007 1167 42 55 10.1016/j.brainres.2007.04.087 17662264

[B32] Panzanelli P Fritschy JM Yanagawa Y Obata K Sassoe-Pognetto M GABAergic phenotype of periglomerular cells in the rodent olfactory bulb J Comp Neurol 2007 502 990 1002 10.1002/cne.21356 17444497

[B33] Pinching AJ Powell TP The neuron types of the glomerular layer of the olfactory bulb J Cell Sci 1971 9 305 345 4108056 10.1242/jcs.9.2.305

[B34] Bertrand N Castro DS Guillemot F Proneural genes and the specification of neural cell types Nat Rev Neurosci 2002 3 517 530 10.1038/nrn874 12094208

[B35] Parras CM Schuurmans C Scardigli R Kim J Anderson DJ Guillemot F Divergent functions of the proneural genes Mash1 and Ngn2 in the specification of neuronal subtype identity Genes Dev 2002 16 324 338 10.1101/gad.940902 11825874 PMC155336

[B36] Hodge RD Kowalczyk TD Wolf SA Encinas JM Rippey C Enikolopov G Kempermann G Hevner RF Intermediate progenitors in adult hippocampal neurogenesis: Tbr2 expression and coordinate regulation of neuronal output J Neurosci 2008 28 3707 3717 10.1523/JNEUROSCI.4280-07.2008 18385329 PMC6671086

[B37] Molyneaux BJ Arlotta P Menezes JR Macklis JD Neuronal subtype specification in the cerebral cortex Nat Rev Neurosci 2007 8 427 437 10.1038/nrn2151 17514196

[B38] Whitman MC Greer CA Adult-generated neurons exhibit diverse developmental fates Dev Neurobiol 2007 67 1079 1093 10.1002/dneu.20389 17565001

[B39] Kele J Simplicio N Ferri AL Mira H Guillemot F Arenas E Ang SL Neurogenin 2 is required for the development of ventral midbrain dopaminergic neurons Development 2006 133 495 505 10.1242/dev.02223 16410412

[B40] Andersson E Jensen JB Parmar M Guillemot F Bjorklund A Development of the mesencephalic dopaminergic neuron system is compromised in the absence of neurogenin 2 Development 2006 133 507 516 10.1242/dev.02224 16396906

[B41] Dellovade TL Pfaff DW Schwanzel-Fukuda M Olfactory bulb development is altered in small-eye (Sey) mice J Comp Neurol 1998 402 402 418 10.1002/(SICI)1096-9861(19981221)402:3<402::AID-CNE8>3.0.CO;2-0 9853907

[B42] Hack MA Saghatelyan A de Chevigny A Pfeifer A Ashery-Padan R Lledo PM Gèotz M Neuronal fate determinants of adult olfactory bulb neurogenesis Nat Neurosci 2005 8 865 872 15951811 10.1038/nn1479

[B43] Kohwi M Osumi N Rubenstein JL Alvarez-Buylla A Pax6 is required for making specific subpopulations of granule and periglomerular neurons in the olfactory bulb J Neurosci 2005 25 6997 7003 10.1523/JNEUROSCI.1435-05.2005 16049175 PMC6724841

[B44] Brill MS Snapyan M Wohlfrom H Ninkovic J Jawerka M Mastick GS Ashery-Padan R Saghatelyan A Berninger B Gotz M A dlx2- and pax6-dependent transcriptional code for periglomerular neuron specification in the adult olfactory bulb J Neurosci 2008 28 6439 6452 10.1523/JNEUROSCI.0700-08.2008 18562615 PMC3844782

[B45] Toresson H Potter SS Campbell K Genetic control of dorsal-ventral identity in the telencephalon: opposing roles for Pax6 and Gsh2 Development 2000 127 4361 4371 11003836 10.1242/dev.127.20.4361

[B46] Yun K Potter S Rubenstein JL Gsh2 and Pax6 play complementary roles in dorsoventral patterning of the mammalian telencephalon Development 2001 128 193 205 11124115 10.1242/dev.128.2.193

[B47] Scardigli R Baumer N Gruss P Guillemot F Le Roux I Direct and concentration-dependent regulation of the proneural gene Neurogenin2 by Pax6 Development 2003 130 3269 3281 10.1242/dev.00539 12783797

[B48] Kohwi M Petryniak MA Long JE Ekker M Obata K Yanagawa Y Rubenstein JL Alvarez-Buylla A A subpopulation of olfactory bulb GABAergic interneurons is derived from Emx1- and Dlx5/6-expressing progenitors J Neurosci 2007 27 6878 6891 10.1523/JNEUROSCI.0254-07.2007 17596436 PMC4917362

[B49] Boutin C Diestel S Desoeuvre A Tiveron MC Cremer H Efficient *in vivo *electroporation of the postnatal rodent forebrain PLoS ONE 2008 3 e1883 10.1371/journal.pone.0001883 18382666 PMC2270900

[B50] Ezeh PI Wellis DP Scott JW Organization of inhibition in the rat olfactory bulb external plexiform layer J Neurophysiol 1993 70 263 274 8395579 10.1152/jn.1993.70.1.263

[B51] Belluscio L Lodovichi C Feinstein P Mombaerts P Katz LC Odorant receptors instruct functional circuitry in the mouse olfactory bulb Nature 2002 419 296 300 10.1038/nature01001 12239567

[B52] Kosaka T Kosaka K Tyrosine hydroxylase-positive GABAergic juxtaglomerular neurons are the main source of the interglomerular connections in the mouse main olfactory bulb Neurosci Res 2008 60 349 354 10.1016/j.neures.2007.11.012 18206259

[B53] Kiyokage E Pan YZ Shao Z Kobayashi K Szabo G Yanagawa Y Obata K Okano H Toida K Puche AC Shipley MT Molecular identity of periglomerular and short axon cells J Neurosci 2010 30 1185 1196 10.1523/JNEUROSCI.3497-09.2010 20089927 PMC3718026

[B54] Seibt J Schuurmans C Gradwhol G Dehay C Vanderhaeghen P Guillemot F Polleux F Neurogenin2 specifies the connectivity of thalamic neurons by controlling axon responsiveness to intermediate target cues Neuron 2003 39 439 452 10.1016/S0896-6273(03)00435-5 12895419

[B55] Srinivas S Watanabe T Lin CS William CM Tanabe Y Jessell TM Costantini F Cre reporter strains produced by targeted insertion of EYFP and ECFP into the ROSA26 locus BMC Dev Biol 2001 1 4 10.1186/1471-213X-1-4 11299042 PMC31338

[B56] De Paola V Arber S Caroni P AMPA receptors regulate dynamic equilibrium of presynaptic terminals in mature hippocampal networks Nat Neurosci 2003 6 491 500 12692557 10.1038/nn1046

[B57] Toth ZE Mezey E Simultaneous visualization of multiple antigens with tyramide signal amplification using antibodies from the same species J Histochem Cytochem 2007 55 545 554 10.1369/jhc.6A7134.2007 17242468

[B58] Lind D Franken S Kappler J Jankowski J Schilling K Characterization of the neuronal marker NeuN as a multiply phosphorylated antigen with discrete subcellular localization J Neurosci Res 2005 79 295 302 10.1002/jnr.20354 15605376

[B59] Chang YC Gottlieb DI Characterization of the proteins purified with monoclonal antibodies to glutamic acid decarboxylase J Neurosci 1988 8 2123 2130 3385490 10.1523/JNEUROSCI.08-06-02123.1988 PMC6569335

