# Supplementary figures and images for: Sequential generation of olfactory bulb glutamatergic neurons by Neurog2-expressing precursor cells
Source: Neural Dev. 2011 Apr 5;6:12. doi: 10.1186/1749-8104-6-12 (PMC3087671; doi:10.1186/1749-8104-6-12)

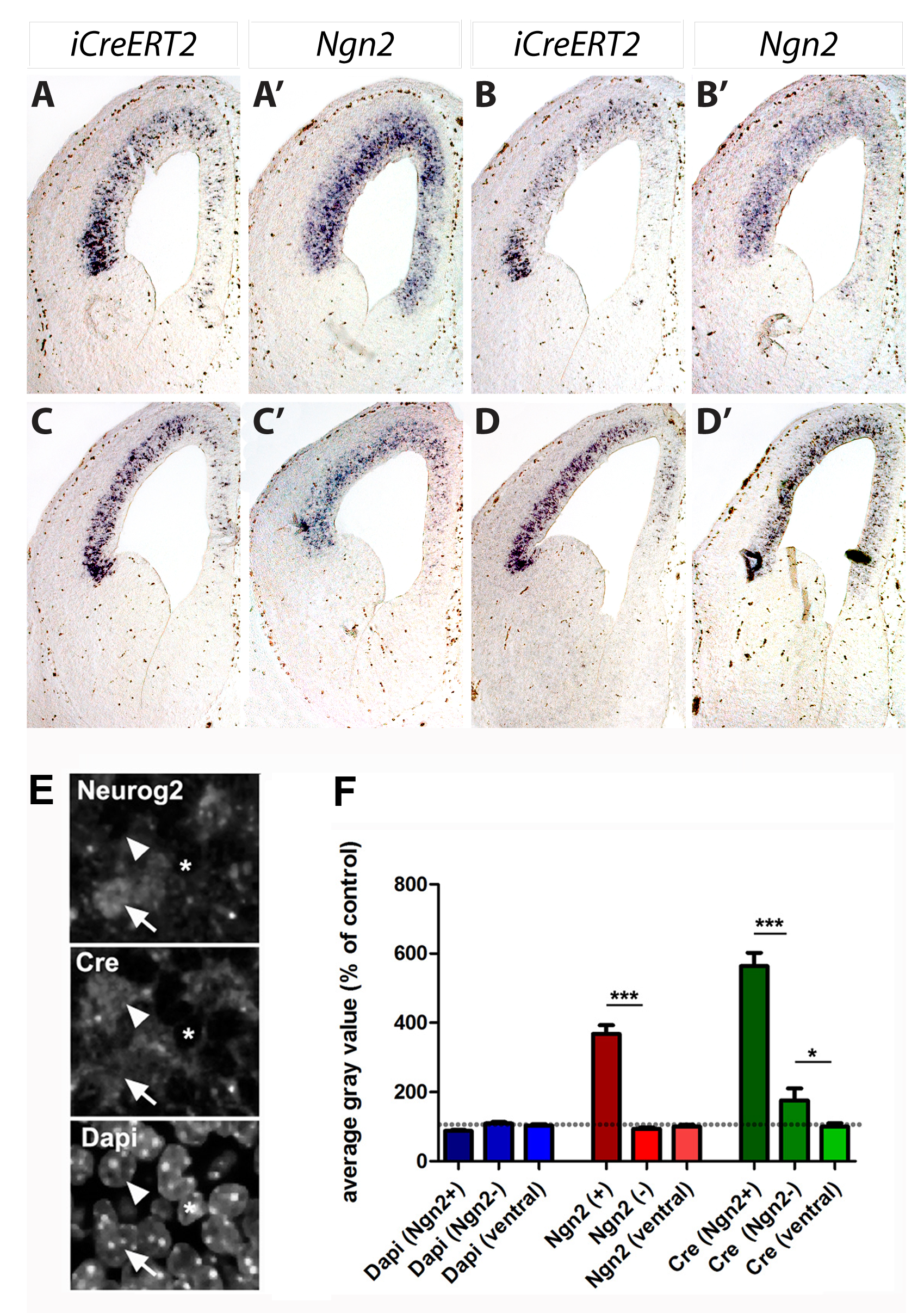

Supplement: Additional file 1 — Figure S1: The expression of iCreERT2 follows Neurog2 expression in the E14.5 mouse telencephalon. (A-D') In situ hybridization showing the expression of iCreERT2 (A,B,C,D) and Ngn2 (A',B',C',D') at different antero-posterior levels of the E14.5 mouse telencephalon (from anterior (A,A') to posterior (D,D') telencephalon. (E,F) Immunodetection of Neurog2 (E, top panel) and iCreERT2 (E, middle panel) in the newborn lateral ventricle reveals co-expression of the two markers in the same cells (DAPI nuclear counterstain; E, bottom panel). Note that while some cells are positive for both Neurog2 and iCreERT2 (arrow), some have downregulated Neurog2 but still express iCreERT2 (arrowhead) due to its longer half-life. Surrounding cells are consistently negative for the two markers (asterisk). Quantifications were performed by densitometry analysis (F). Grey values for DAPI, Neurog2 and iCreERT2 were measured in randomly selected DAPI(+) cells in the dorsal and ventral walls of the lateral ventricles of three Neurog2iCreERT2 animals (n > 50 cells per animal). Note the clear detection of iCreERT2 in the Neurog2(+) cell fraction. Note that iCreERT2 can also be detected in some Neurog2(-) cells, presumably cells that have downregulated Neurog2 but still express iCreERT2 due to its longer half-life. [file 1749-8104-6-12-S1.TIFF]

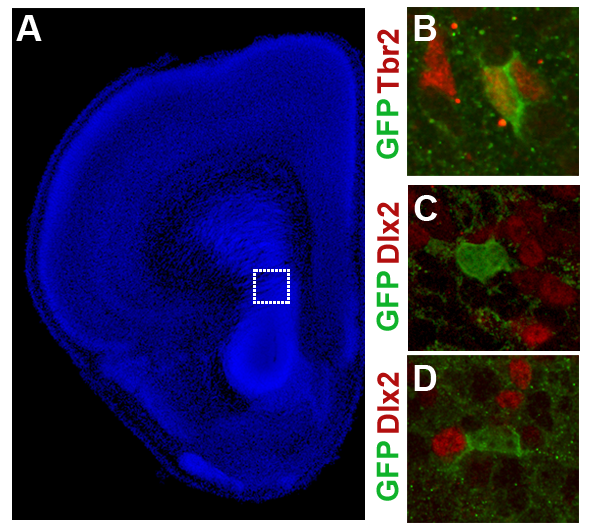

Supplement: Additional file 2 — Figure S2: Short-term fate mapping of the Neurog2 progeny in the Neurog2iCreERT2 mice. Neurog2iCreERT2 mice were crossed with RosaYFP reporter mice. Animals were injected with tamoxifen at P0 and scarificed 24 hours later. (A-D) Recombined cells could be observed at the transition of the lateral ventricle with the RMS (A) (the doted box on the DAPI counterstained cross-section indicates the approximate location of the GFP(+) cells shown in (B-D)). GFP immunostaining showed co-localization with Tbr2 (B) (100%, n = 32) but not with Dlx2 (C, D) (0%, n = 15), confirming the faithful expression of the Cre recombinase and the efficient labeling of the Neurog2 cell lineage. [file 1749-8104-6-12-S2.TIFF]

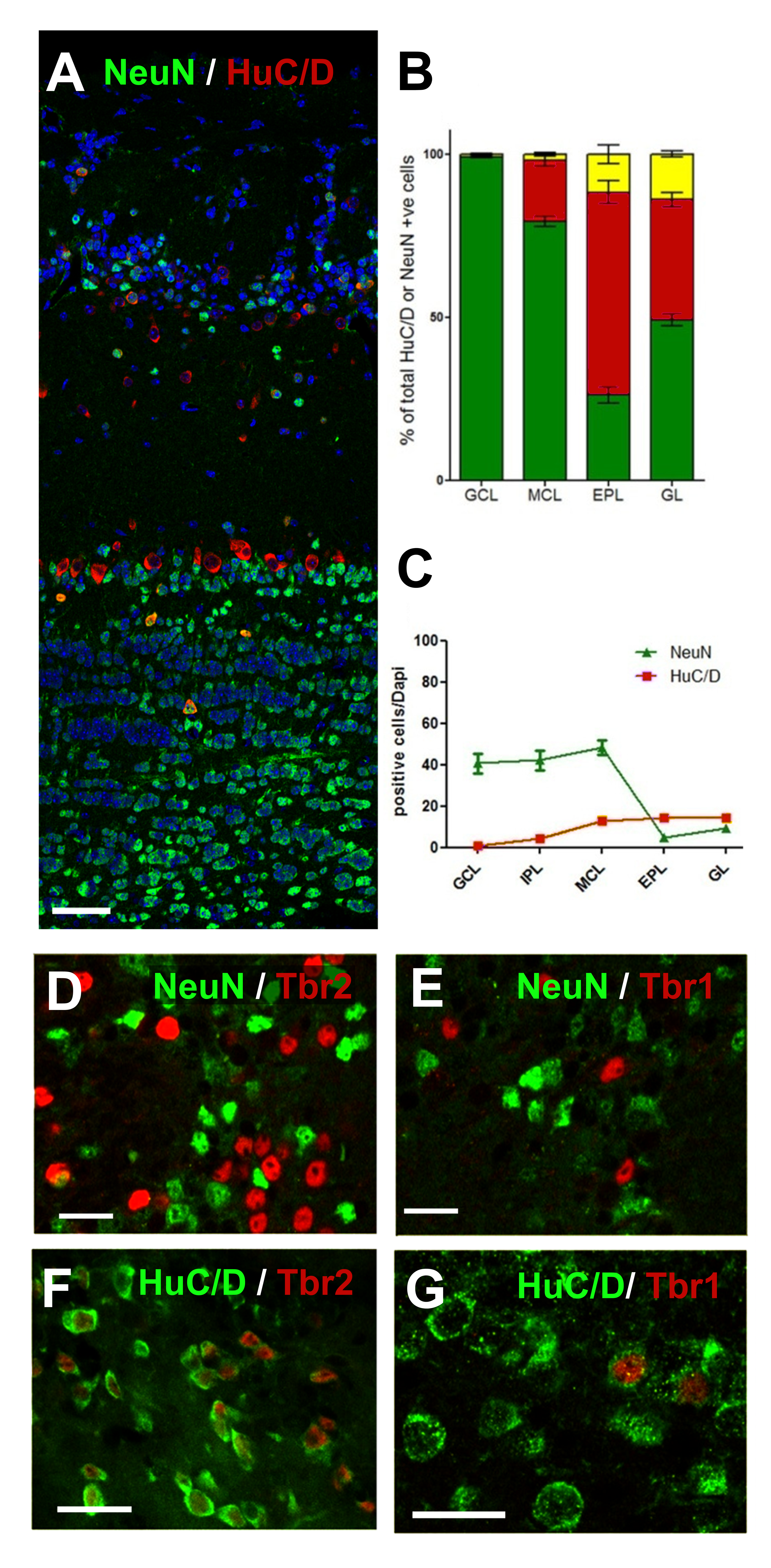

Supplement: Additional file 3 — Figure S3: Neuronal phenotype of Tbr1/Tbr2-positive cells. (A,B) Sequential double immunostaining for NeuN (green) and HuC/D (red) reveals different degrees of co-localization (yellow) of the two markers throughout the OB layers. (C) Percentage of DAPI-positive cells expressing either NeuN or HuC/D in each OB layer. (D-G) Double immunostaining of Tbr2 or Tbr1 with either NeuN or HuC/D. NeuN is excluded from the Tbr1- and Tbr2-positive cells while HuC/D preferentially labels the two OB glutamatergic neuron populations. Scale bars: 100 μm in (A); 20 μm in (D-G). [file 1749-8104-6-12-S3.TIFF]

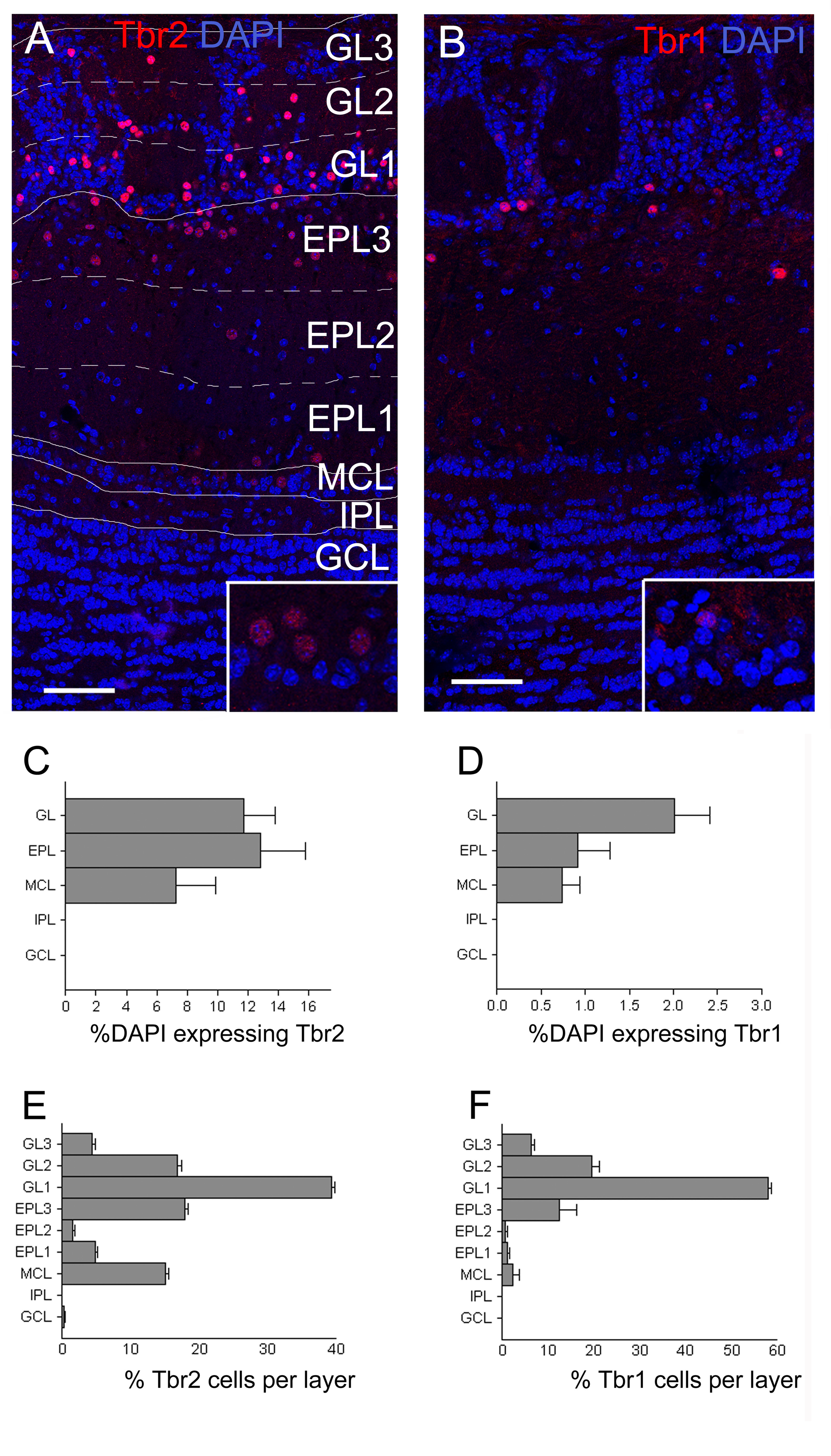

Supplement: Additional file 4 — Figure S4: Distribution of Tbr1- and Tbr2-positive neurons in the mouse olfactory bulb. (A,B) Overview showing the distribution pattern of Tbr2 and Tbr1 immunoreactivity in the adult OB. (C-F) Graphs showing the quantification of the distribution of Tbr2 and Tbr1 in the different OB cell layers. [file 1749-8104-6-12-S4.TIFF]

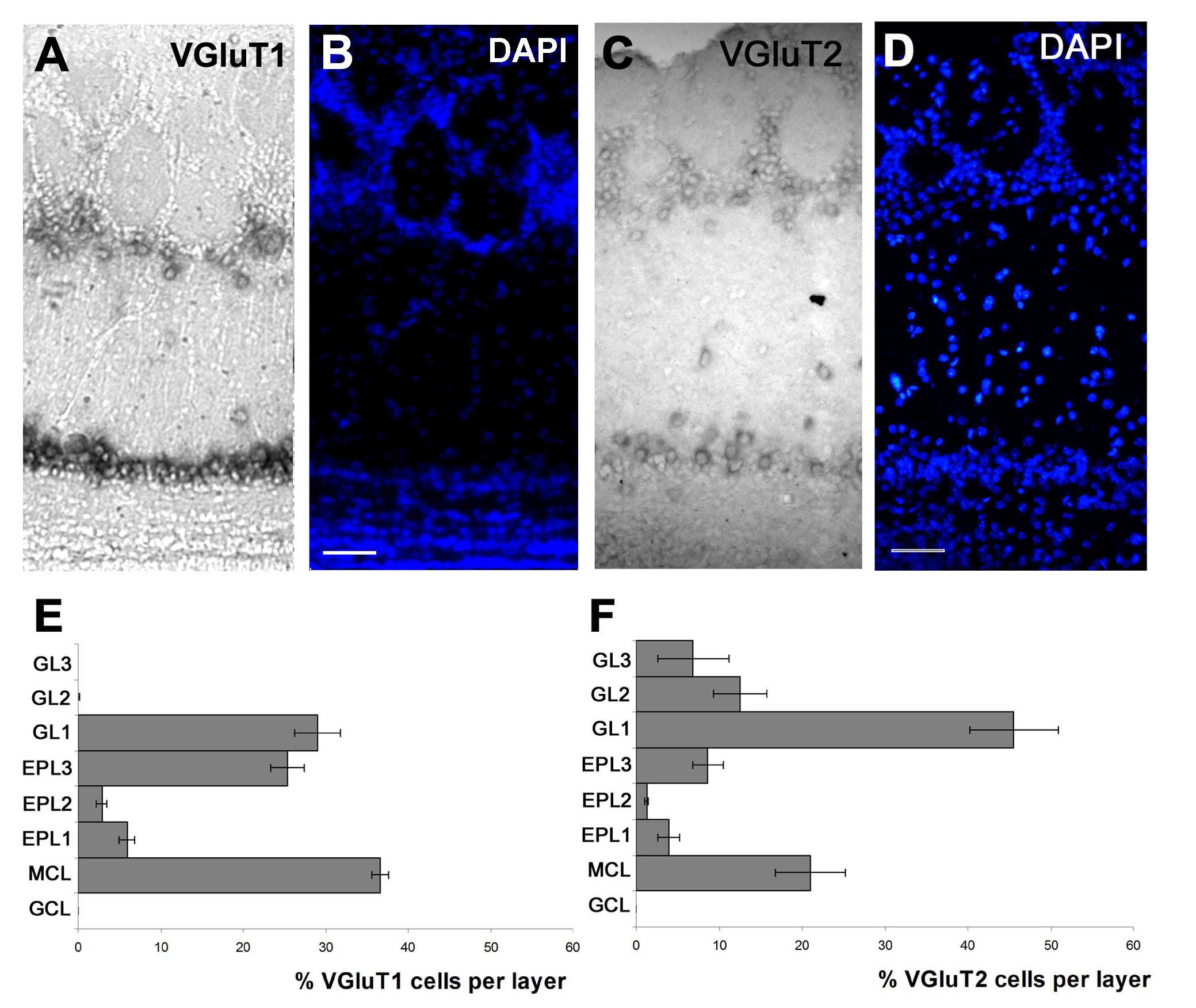

Supplement: Additional file 5 — Figure S5: Distribution of VGlut1- and VGlut2-positive neurons in the mouse olfactory bulb. (A-D) Distribution pattern of VGluT2 and VGlut1 in the adult OB. VGluT1 in situ hybridization shows a strong signal in the MCL and a weaker signal at the boundary of the EPL and GL (A). VGluT2 mRNA is found both in the MCL and throughout the GL (C). DAPI counterstaining (B, D) allows the visualization of the distinct OB layers. (E-F) Graphs showing the quantification of the distribution of VGlut1 and VGlut2 in the different OB cell layers. When we considered the VGluT-positive cells as a proportion of DAPI(+) cells, we found a similar distribution. VGluT1(+) cells accounted for around 14% of DAPI(+) cells in the MCL, EPL1, and EPL3, with a smaller proportion in EPL2 and GL1. VGluT2(+) cells were more widely distributed between the layers. In the GL as a whole, VGluT2 accounted for 15% of DAPI(+) cells, 19% in the MCL, and a lower proportion in the EPL. [file 1749-8104-6-12-S5.TIFF]

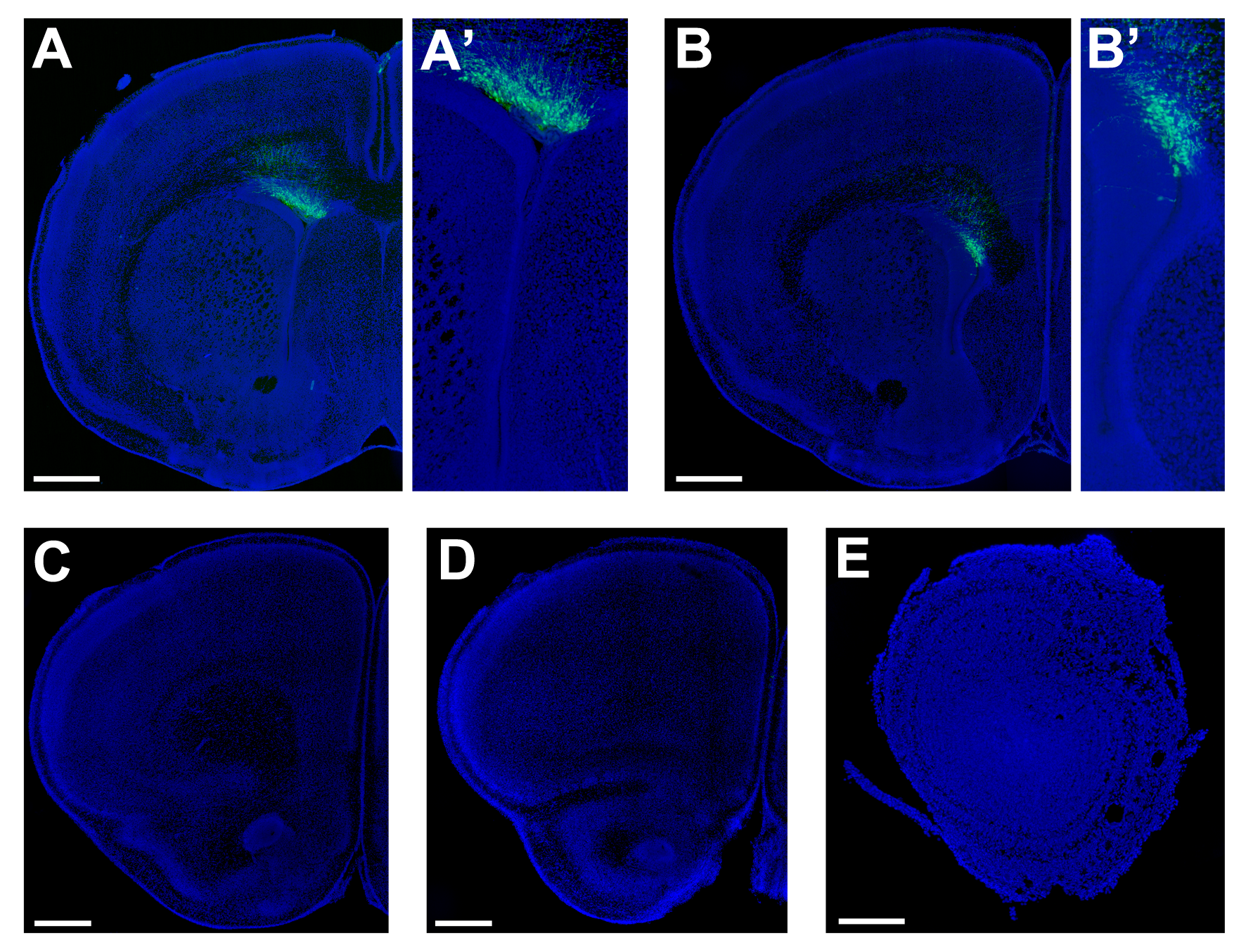

Supplement: Additional file 6 — Figure S6: Local expression of GFP after dorsal electroporation of the lateral ventricle. (A-E) Native GFP expression observed 48 hours after electroporation of a GFP expression plasmid in the dorsal wall of the lateral ventricle of a newborn mouse at different caudo-rostral levels (from caudal (A) to rostral (E, showing the OB)). DAPI was used as a nuclear counterstain (blue). Scale bar: 1 mm in (A-D); 500 μm in (E). [file 1749-8104-6-12-S6.TIFF]
